# Drinking waters contamination by per‐ and poly‐fluoroalkyl substances of the anthropized area of Fos‐Berre, France

**DOI:** 10.1002/jeq2.70241

**Published:** 2026-09-21

**Authors:** M. Dijoux, A. Piram, S. Augy, A. Austruy, A. Souloumiac, L. De Jong, E. Gonzales‐Camoin, J. Dron, P. Chamaret, X. Moreau, S. Gori, P. Wong‐Wah‐Chung, P. Doumenq

**Affiliations:** ^1^ Société ABO‐ERG Environnement Vitrolles France; ^2^ LCE Aix Marseille University Aix‐en‐Provence France; ^3^ Institut Écocitoyen pour la Connaissance des Pollutions Fos‐sur‐Mer France; ^4^ IMBE, CNRS, IRD Aix Marseille University, Avignon University Marseille France

## Abstract

Per‐ and poly‐fluoroalkyl substances (PFAS) contamination and geographical distribution were studied in drinking water from the industrial harbor of Fos‐Berre, a highly industrialized area in southern France. An analytical method for 38 PFAS was developed, using large volume injection ultra‐high performance liquid chromatography‐tandem mass spectrometry. To minimize the risk of contamination during preparation, samples were directly injected onto a SCIEX Triple Quad 7500 system after dilution in acidified methanol. Method limits of quantification ranged from 0.081 to 2.2 ng L^−1^, except for 8:2 fluorotelomer unsaturated carboxylic acid and perfluoropentanoic acid (5.4 ng L^−1^). The method was applied to 28 tap waters and two groundwater from 28 stations on the Crau groundwater table and Durance River. Eighteen PFAS were quantified in at least one sample, with concentrations from 0.14 for linear perfluorooctane sulfonic acid (PFOS) to 62.85 ng L^−1^ for 6:2 fluorotelomer sulfonic acid. Short‐chain carboxylic acids (perfluorocarboxylic acids) and sulfonic acids (perfluorosulfonic acids) were the most frequently quantified, with perfluorohexanoic acid showing the highest frequency (73%), followed by perfluorooctane carboxylic acid, branched PFOS, branched perfluorooctane hexanoic acid (PFHxS), linear PFOS and linear PFHxS. Total PFAS ranged from <0.081 to 126 ng L^−1^ and the sum of the 20 regulated PFAS remained below the 2020/2184 drinking water directive limit of 100 ng L^−1^ except for one sample (119 ng L^−1^). Contamination levels and profiles varied across sites, reflecting catchment location, surrounding industrial activities, and groundwater flow, with higher concentrations detected downstream.

## Core Ideas

- Thirty‐eight per‐ and poly‐fluoroalkyl substances (PFAS) were investigated in 30 water samples.
- Eighteen PFAS were quantified.
- Perfluorohexanoic acid (PFHxA), perfluorooctane carboxylic acid (PFOA), branched (Br)‐perfluorooctane sulfonic acid (PFOS), Br‐perfluorooctane hexanoic acid (PFHxS), linear (L)‐PFOS, and L‐PFHxS were found in >50% of samples.
- Short‐chain perfluorocarboxylic acids (PFCAs) and perfluorosulfonic acids (PFSAs) were the most frequently quantified PFAS.
- One sample exceeded the European Directive of 100 ng L^−1^ for ∑ 20 PFAS.

## Plain Language Summary

Per‐ and poly‐fluoroalkyl substances (PFAS) are a group of thousands of human‐made chemicals with special properties that have led to their use in almost all industries. They can enter the environment during production and use, where they stay for a long time and harm ecosystems even at very low levels. Once released, PFAS can contaminate groundwater, which may be used to produce tap water. This article describes the development of a scientific strategy from sampling to analysis, to investigate the presence of PFAS in some groundwaters and tapwaters from south‐east of France, at concentrations as low as 1 ng L^−1^. Samples were collected in several private homes thanks to a local citizen participative science network. Eighteen out of 38 molecules investigated have been quantified and exceed, for one sample, the European drinking water directive limit. Contamination levels and profiles varied across sites, reflecting catchment location and surrounding industrial activities. Highest concentrations were detected downstream.

**Abbreviations**
AFFF: aqueous film‐forming foams
CGWT: Crau groundwater table
CRM: certified reference material
DWD: drinking water directive
EFSA: European Food Safety Authority 
FoA: formic acid
GW: raw groundwater
LQ: limit of quantification
MQL: method quantification limit
PCI: classified installations for environmental protection
PFAS: per‐ and poly‐fluoroalkyl substances
PFCAs: perfluorocarboxylic acids
PFSAs: perfluorosulfonic acids
QC: quality control
TW: tap water
TWI: tolerable weekly intake 

## 1 INTRODUCTION

Per‐ and poly‐fluoroalkyl substances (PFAS) comprise between 4730 (OECD, 2018) and 14,735 (U.S. EPA, 2022) partially or fully fluorinated aliphatic compounds, differing by their functional groups (Buck et al., 2011; OECD, 2021) and in the perfluorinated chain length. Carboxylic acids (perfluorocarboxylic acids [PFCAs]) with chain length above seven carbons (above six for sulfonic acids—perfluorosulfonic acids [PFSAs]) are called “long‐chain” PFAS, while those with fewer are “short‐chain,” and those with two to three carbons “ultra short.” Synthesized since the 1940s for their water and grease repellence, heat resistance and nonstick properties (3M, 1999), PFAS are used in firefighting foams, electronics, food contact materials, and textiles (Glüge et al., 2020). During production and use, they are released into air, wastewater, solid waste, and landfills (Dauchy et al., 2017). Their amphiphilic nature and persistence—especially for PFCAs and PFSAs—lead to transfer and accumulation in all environmental compartments (Nakayama et al., 2019): air, soils (Brusseau et al., 2020), sediments, waters, drinking water (Boiteux et al., 2012; Teymoorian et al., 2023), crops, and livestock, resulting in widespread exposure of all forms of life. Despite active research, remediation remains costly and energy intensive (Fang et al., 2024).

Their ubiquitous presence in environmental matrices raises public health concerns due to their potentially significant toxicity. In 2020, the European Food Safety Authority (EFSA) identified association between PFAS absorption and increased risks of thyroid dysfunction, hypercholesterolemia, cancer, and adverse reproductive outcomes. Accordingly, EFSA established a tolerable weekly intake (TWI) of 4.4 ng kg^−^^1^ body weight (bw) for the combined exposure to perfluorooctane sulfonic acid (PFOS), perfluorooctane carboxylic acid (PFOA), perfluorooctane hexanoic acid (PFHxS), and perfluorononanoic acid (PFNA) (EFSA CONTAM Panel et al., 2020). More recently, the French Agency for Food, Environmental and Occupational Health and Safety defined long‐term toxicological reference values for perfluorobutanoic acid (PFBA) (20 µg kg_bw_^−1^ j^−1^), perfluorohexanoic acid (PFHxA) (20 µg kg_bw_^−1^ j^−1^), and 6:2 fluorotelomer sulfonic acid (FTS) (0.33 µg kg_bw_^−1^ j^−1^) (ANSES, 2025). Furthermore, the European Drinking Water Directive (DWD) (2020/2184; European Parliament, 2020) established a regulatory limit for the sum of 20 PFAS (10 PFCAs and 10 PFSAs), to be enforced from 2026 (see detailed list in Table S1) (European Parliament, 2020).

Studying PFAS behavior in the environment requires highly sensitive analytical methods capable of detecting ultra‐trace concentrations (ng L^−^^1^). Given the high risk of sample contamination and compound losses through adsorption or volatilization, rigorous handling procedures are essential, with minimized preparation steps to reduce these risks.

This study aimed to assess PFAS occurrence in drinking water from the Gulf of Fos and the Berre Lagoon (Fos‐Berre area), a heavily industrialized region in southern France (“Région Sud”) that hosts no PFAS production facilities. An optimized analytical protocol was therefore developed for 38 PFAS (see Section 2.2) and applied to 28 tap water (TW) samples mainly supplied from collective water catchments of the Crau groundwater table (CGWT), and two groundwater samples.

## 2 MATERIALS AND METHODS

### 2.1 Sampling site description

The Crau Plain, located west of the Berre Lagoon and north of the Gulf of Fos in southern France, covers approximately 550 km^2^ and hosts the CGWT, a major strategic freshwater resource for drinking water, agriculture, and industry. The CGWT supplies about 270,000 inhabitants in 13 municipalities via 18 public well fields and numerous private wells. Approximately 70% of its recharge originates from traditional irrigation of Crau hay meadows, with the remaining 30% derived from precipitation (SYMCRAU, 2019), which produces an artificial annual cycle characterized by high water levels in summer and low levels in winter. In the absence of an overlying impermeable layer, the aquifer is highly vulnerable to contamination from surrounding human activities. To characterize the influence of anthropogenic pressures, the study area included rural, urban (about 10% of the surface), and industrial settings. In the northern and northwestern sectors, agricultural practices may impact groundwater quality through the use of fertilizers and pesticides. The southern and eastern sectors are affected by emissions from the chemical, petrochemical, and metallurgical activities of the Fos‐sur‐Mer industrial harbor, the largest industrial port complex in France, and by waste incineration plants, extensive oil and gas pipeline networks, a military air base, and intense maritime, road, and air traffic. Additional sources of contamination include fuel storage, aircraft cleaning with products containing fluorinated surfactants, and the frequent use of aqueous film‐forming foams (AFFF) on military and airport facilities (SYMCRAU, 2024). Although no PFAS production plants are reported in the area, seven classified installations for environmental protection located directly above the CGWT are subjected to PFAS monitoring in industrial effluents according to the French Decree of June 20, 2023.

**Core Ideas**

- Thirty‐eight per‐ and poly‐fluoroalkyl substances (PFAS) were investigated in 30 water samples.
- Eighteen PFAS were quantified.
- Perfluorohexanoic acid (PFHxA), perfluorooctane carboxylic acid (PFOA), branched (Br)‐perfluorooctane sulfonic acid (PFOS), Br‐perfluorooctane hexanoic acid (PFHxS), linear (L)‐PFOS, and L‐PFHxS were found in >50% of samples.
- Short‐chain perfluorocarboxylic acids (PFCAs) and perfluorosulfonic acids (PFSAs) were the most frequently quantified PFAS.
- One sample exceeded the European Directive of 100 ng L^−1^ for ∑ 20 PFAS.

**FIGURE 1:**
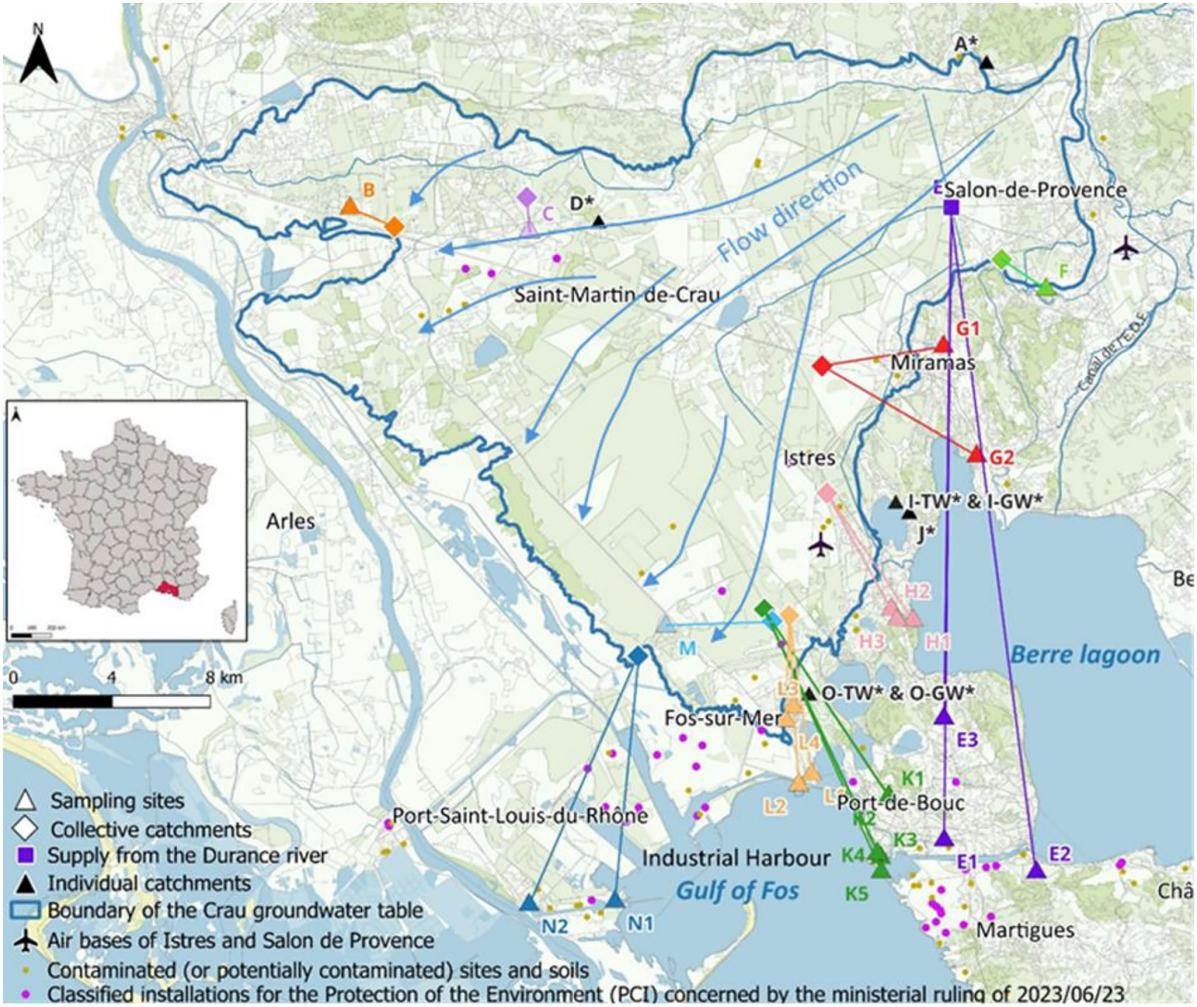
Map of the 30 water sampling sites (triangles). Colored triangles are linked by lines to their collective catchments (from drinking water treatment plants) (diamonds for the Crau groundwater table (CGWT) and square for the Durance River) while black triangles indicate private catchments (private well from individual household). Sample identity: A–O, with * indicating drinking water supplied by the private catchments. Groundwater flow directions were obtained from the SYMCRAU. GW, raw groundwater; TW, tap water.

The study area and sampling locations are shown in Figure 1. In June 2024, 30 water samples (mainly TW) were collected from 28 sites. Sampling points (A–O) are represented by triangles and are named according to their associated catchments, which are shown as diamonds for CGWT and as a square for the Durance River; black triangles indicate private wells, and an asterisk identifies those used for drinking water supply.

All 28 TWs were collected from private households. Three TWs were supplied from one collective catchment on the Durance River, 20 TWs from nine collective catchments on the CGWT, and five TWs from individual water catchments located in the vicinity of the CGWT (except for site D*). For two of the five sites using individual water catchment, the groundwater (used as TW source) was also collected, from pipes in the gardens, to evaluate the efficiency of the domestic treatments.

Groundwater analysis was not part of the initial experimental design. It was performed only to compare baseline PFAS concentrations with those measured in TW after treatment using domestic purification systems, with the aim of evaluating the removal efficiency of these processes. Additional information on catchment type and the presence of treatment or filtration is provided in Table 1.​

**TABLE 1:** Information on the water samples, catchments (A–O), household individual, and collective treatments.

Sample ID	Water origin	T (°C)	Samples coord.	Household water treatment system	Associated water catchment and ID		Catchment coord.	Catchment type	Collective water treatment system
A^a^	Groundwater at the edge of the CGWT	17.9	43.692; 5.045	No	Individual	A^a^	43.692;5.045	Individual	/
B	CGWT	21.9	43.645; 4.723	No	St Hippolyte	B	43.637133; 4.750683	Collective	Chlorine gas
C	CGWT	24.1	43.635; 4.813	No	Val Boisé	C	43.641855; 4.809606	Collective	Chlorine gas
D^a^	CGWT	22.4	43.673; 4.892	No	Individual	D^a^	43.637; 4.847	Individual	/
E1	Durance	22.3	43.409; 5.013	No	Durance	E	43.64023; 5.02513	Collective	Ozone and/or chlorine
E2		22.5	43.396; 5.059	No					
E3		21.5	43.453; 5.015	No					
F	CGWT	21.1	43.609; 5.071	No	Mary Rose	F	43.620012; 5.048790	Collective	No information
G1	CGWT	20	43.589; 5.019	No	Puits des Canaux Jumeaux	G	43.605493; 4.958637	Collective	Chlorine gas
G2		22.9	43.549; 5.035	No					
H1	CGWT	20.4	43.490; 5.001	20 µm sediment filter	La Caspienne	H	43.538622; 4.941626	Collective	Chlorine gas
H2		23.4	43.494; 4.990	No					
H3		24.1	43.490; 4.992	No					
I‐TW^a^	Groundwater at the edge of the CGWT	23.8	43.532; 4.993	10 µm filter, softener, osmosis with activated carbon, U.V.	Individual	I^a^	43.532; 4.993	Individual	/
I‐GW^a^		16.6	43.532; 4.993	No					
J^a^	Groundwater at the edge of the CGWT	21.3	43.528; 5.000	No	Individual	J^a^	43.528; 5.000	Individual	/
K1	CGWT	22.4	43.425; 4.986	No	Des Tapies	K	43.486058; 4.920420	Collective	Direct chlorination
K2		21.8	43.425; 4.986	No					
K3		21.5	43.403; 4.981	No					
K4		23.2	43.404; 4.980	Old salt softener					
K5		24.7	43.398; 4.981	No					
L1	CGWT	25.8	43.434; 4.948	No	Fanfarigoule‐BA	L	43.484885; 4.948613	Collective	Direct chlorination
L2		22.3	43.430; 4.941	No					
L3		20.5	43.459; 4.940	No					
L4		24.7	43.454; 4.935	No					
M	CGWT	22.8	43.490; 4.877	No	Ventillon	M	43.478763; 4.926804	Collective	No information
N1	CGWT	20.9	43.389; 4.848	No except for hot water + asbestos pipe	La Pissarote	N	43.490395; 4.846588	Collective	No information
N2		21.5	43.389; 4.805	Softener, cotton					
O‐TW^a^	Groundwater at the edge of the CGWT	17.9	43.463; 4.947	Taste filter, textile cartridge (cotton), glyphosate filter (crystals), carbon filter	Individual	O^a^	43.463; 4.947	Individual	/
O‐GW^a^		17.6	43.463; 4.947	No					

*Note*: Sample coord. and Catchment coord. correspond to the latitude and longitude coordinate of sampling sites and the corresponding water catchment. /: no information.

Abbreviations: CGWT, Crau groundwater table; GW, raw groundwater; T (°C), temperature of the water sampled; TW, tap water, GW = raw groundwater.

^a^ Drinking water supplied by the private catchments.

Previous investigations in the Fos–Berre region have documented trace elements and organochlorine contaminants in soils (Austruy et al., 2021), as well as PFAS in drinking water (ARS, 2025b) and in marine biota, although quantitative PFAS data for this area remain limited. The collection of tap water and groundwater samples in this study was made possible through collaboration with volunteers from the Eco Citizen Institute for the Knowledge of Pollution within the VOCE citizen science network (Volunteers for the Citizens’ Observation of the Environment).

### 2.2 Chemicals and standards

Thirty‐five PFAS regulated by either the DWD 2020/2184, the ISO Standard 21675, or the French ministerial ruling for classified installations for environmental protection (PCI) were purchased: 13 PFCAs (C_3_–C_18_), 10 PFSAs (C_4_–C_13_), two FTS, two perfluoroalkyl ether carboxylic acids, one perfluoroalkyl ether sulfonic acid, one fluorotelomer unsaturated carboxylic acid (FTUCA), one polyfluoroalkyl phosphoric acid diester, three perfluoroalkyl sulfonamides (FASAs), and two perfluoroalkyl sulfonamido acetic acids. For that, a 2 mg L^−1^ mixture of 29 PFAS in methanol was purchased from Accustandard (ISO 21675 R1) (TechLab), and other PFAS were purchased as individual methanolic solutions of 2, 50, or 100 mg L^−1^ from Wellington laboratories (B.C.P. Instruments), Accustandard, Cambridge Isotope Laboratories Inc (CIL) (TechLab), and Chiron AS (BCP Instruments). PFOS, PFHxS, and N‐ethyl perfluorooctane sulfonamido acetic acid (N‐EtFOSAA) standards contained a mixture of linear (L‐) and branched (Br‐) isomers, making a list of 38 PFAS. Seven isotopically labeled PFAS were purchased from CIL as two commercial mixtures (ES‐5610A: ^13^C_8_‐PFOA, ^13^C_8_‐potassium perfluoro‐1‐octanesulfonate [PFOS], and d_3_‐N‐methyl perfluorooctane sulfonamido acetic acid [d_3_‐NMeFOSAA] at respective concentrations of 1, 3, and 4 mg L^−1^; and ES‐5632: ^13^C_3_‐sodium tetrafluoro‐2‐(heptafluoropropoxy)propanoate [^13^C_3_‐HFPO‐DA], ^13^C_6_‐sodium perfluoro‐n‐hexanoate [^13^C_6_‐PFHxA], ^13^C_9_‐sodium perfluoro‐n‐decanoate [^13^C_9_‐PFDA], and d_5_‐NEtFOSAA at respective concentrations of 1, 1, 1, and 4 mg L^−1^) and used as internal standard (IS) stock solutions. In the rest of the document, we will refer to the acidic form of the compounds. The full list of compounds with their chemical names and abbreviations can be found in Table S1. The standard and isotopically labeled standard solutions were prepared by dilution in methanol and were stored in polypropylene vials at 4°C or −20°C depending on the supplier's recommendation.

ULC‐MS grade methanol, water, and ammonium acetate were purchased from Biosolve Chimie. Formic acid (FoA) was obtained from Fisher Scientific SAS. Evian mineral bottled water was used for calibration and quality control (QC) solutions, after assessing the absence of PFAS contamination. Polypropylene vials were purchased from Agilent Technologies France with silicon caps.

A 250 g L^−1^ ammonium acetate (Biosolve Chimie) solution was prepared as preservative solution.

### 2.3 Sample collection

Samples were collected within 2 week in June 2024 using 250‐mL high‐density polyethylene bottles prewashed with ultrapure water and methanol to minimize PFAS contamination. At each site, taps were flushed for 5 min to stabilize temperature and flow and to remove any tap‐accumulated contamination. Bottles were then rinsed three times with site water before collecting approximately 250 mL. Immediately after collection, 1 mL of ammonium acetate preservative solution was added to each sample to neutralize residual free chlorine and prevent degradation, in accordance with USEPA Method 533 (US EPA, 2019). Field blanks were prepared by filling high‐density polyethylene bottles with ultrapure water on site and exposing them to the sampling environment to assess potential contamination during collection and transport.

To minimize contamination, waterproof clothing and food packaging were avoided during sampling, non‐powdered nitrile gloves were worn, samples were placed individually in labeled plastic bags, and no permanent markers were used. All fluoropolymer‐containing equipment was replaced with alternative materials. Samples were transported to the laboratory within 24 h in a cooler containing natural ice. Upon arrival, raw samples were stored at −20°C and analyzed within 2 weeks of collection.

### 2.4 Sample preparation

In this study, samples were prepared by direct injection after dilution with methanol to limit matrix effects, contamination (cross‐contamination, materials, and reagents), and analyte losses by adsorption or volatilization during preconcentration.

Polypropylene and polyethylene are generally recommended as PFAS container materials (ISO 21675, 2019; US EPA, 2019), although glass is also frequently used (Zenobio et al., 2022). Some studies report negligible or lower PFAS sorption to polypropylene compared with glass, while others show stronger sorption to polypropylene (Woudneh et al., 2019; Zenobio et al., 2022). Adsorption depends on matrix composition and adding methanol increases PFAS solubility and reduces sorption (Mancini et al., 2023), especially for long‐chain PFAS. With polypropylene vials, a 50:50 MeOH/H_2_O ratio gave poor recoveries for the longest‐chain PFCA (perfluorooctadecanoic acid). Testing methanol contents from 50% to 99% showed that FASAs exhibited peak asymmetry above 70% methanol. A MeOH/H_2_O ratio of 60:40 (v/v) with 0.1% FoA was therefore selected to improve long‐chain PFAS solubility without excessively increasing limits of quantification (LQs).

Upon arrival at the laboratory, samples were prepared immediately. After thawing at room temperature, 250 mL samples were homogenized by shaking, and 400 µL aliquots were transferred into polypropylene autosampler vials. Each aliquot was diluted with 600 µL methanolic FoA to reach a final MeOH/sample ratio of 60:40 (v/v) with 0.1% FoA, then spiked with ISs. Polypropylene vials with silicone septa were used to minimize contamination. Prepared samples were stored frozen until analysis to limit transformation of polyfluoroalkyl substances, since biodegradation can occur at ambient temperature (Woudneh et al., 2019). Polyfluoroalkyl substances that can transform into more stable PFAS such as PFCAs and PFSAs are frequently called precursors.

### 2.5 Analytical methods

The analytical method was developed to quantify 38 PFAS in TW, selected from ISO 21675, the French PCI regulation, and the EU DWD 2020/2184. The 38 target PFAS were analyzed using an Agilent 1290 Infinity II UPLC coupled to a SCIEX Triple Quad 7500 tandem mass spectrometer (ultra‐high performance liquid chromatography‐tandem mass spectrometry). Samples (100 µL) were injected onto a Luna Omega PS C18 column (3 µm, 100 Å, 100 mm × 3.0 mm, Phenomenex) protected by a UHPLC ZORBAX Eclipse Plus C18 guard column (2.1 mm × 5 mm, 1.8 µm, Agilent). To prevent background contamination coming from the analytical system, a Gemini C18 delay column (5 µm, 110 Å, 50 mm × 3.0 mm, Phenomenex) was installed between the solvent mixer and the injector. The needle was washed for 10 s between injections with 50:50 MeOH/H_2_O to limit carryover.

The mobile phase consisted of water with 2 mM ammonium acetate (A) and methanol (B). The gradient at 600 µL min^−^^1^ was 10% B for 0.5 min, 10%–50% B over 1 min, 50%–95% B over 6.5 min, 95% B for 4 min, and then re‐equilibration to 10% B over 2.5 min. The column temperature was 40°C. Detection and quantification were performed by MS/MS in scheduled multiple reaction monitoring (sMRM) mode using two transitions per compound, with the most intense transition for quantification and the second for confirmation. Ionization was operated in negative mode with curtain gas, ion source gas 1, and ion source gas 2 set to 40, 50, and 80 psi, respectively, and a source temperature of 350°C.

The ion spray voltage was set at 1500 or 2500 V depending on the compound. Optimization from 1000 to 3000 V showed that long‐chain PFAS signals increased with voltage and plateaued at 2500 V, whereas lower molecular weight compounds exhibited maximum sensitivity at 1500 V. PFAS transitions are described in Table S1. Linear and branched isomers of PFHxS, PFOS, and N‐EtFOSAA were quantified separately by integrating their individual chromatographic peaks. Ten calibration levels from 0.1 to 250 ng L^−^^1^ (0.1, 0.5, 1, 2.5, 5, 10, 25, 50, 100, and 250 ng L^−^^1^) and three QCs (1, 5, and 50 ng L^−^^1^) were prepared in ULC‐MS methanol/Evian water (60:40, v/v) to correct for potential matrix effects in TW (Grüning, 2022), after assessing the absence of PFAS contamination. Quantification of the 38 PFAS was performed using an internal calibration with seven isotope‐labeled standards. For analytes lacking labeled analogues, ISs were attributed following their properties (see Table S1).

### 2.6 Quality assurance and QC

Due to the widespread use of PFAS in everyday objects and laboratory equipment, stringent precautions were implemented to prevent sample contamination during collection and preparation. All fluoropolymers (e.g., Teflon) were avoided throughout the process. One field blank per sampling site was prepared and analyzed, following the same preparation steps as water samples, to assess procedural background and contamination. Solvent blanks (MeOH/H_2_O 60:40 v/v with 0.1% FoA) were run before and periodically within each batch to evaluate instrumental background and carryover.

Method precision, accuracy, and instrument performance were verified for each analytical run through regular analysis of QCs at three concentration levels. Accuracy was further assessed using water certified reference material (CRM IRMM‐428) and by duplicate analysis of five samples at an accredited laboratory. Data acquisition was performed with SCIEX OS software (v. 3.4.0). PFAS were identified based on retention time (±10 s), the presence of at least two selected MRM transitions, and ion abundance ratios (quantitative/qualitative transition) within 30% of acceptable differences.

Seven isotopically labeled PFAS served as ISs for the 38 target analytes, selected based on structural similarity and retention time. Quantification employed quadratic regression with 7–10 calibration levels from 0.1 to 250 ng L^−^^1^. Accuracy criteria were set at ±20% for standards and ±30% for QCs. LQs were defined as the lowest calibration level yielding a signal‐to‐noise ratio of 10.

To account for potential PFAS background contamination arising from the overall preparation and analytical procedure, despite the precautions taken, we quantified its contribution to each sample's signal. This was achieved by measuring PFAS concentrations in field blanks and calculating the ratio of the PFAS concentration in the blank to that in the corresponding sample. Samples were subsequently classified info three categories depending on the blank ratios value: quantifiable, detectable and non‐exploitable. The blank signal was subtracted from the sample when it was below 25% of the sample signal. For a percentage of blank between 25% and 50% of the sample signal, PFAS were considered as detected but not quantifiable (identified as DET). Results were considered non exploitable and therefore discarded when blank values exceeded 50% (identified as NE).

## 3 RESULTS AND DISCUSSION

### 3.1 Method performance parameters and data analysis

All compounds had determination coefficient of their calibration quadratic curves *R* ^2^ above 0.99. Among the 38 compounds, method quantification limits (MQLs) ranged from 0.081 for Br‐PFOS to 5.4 ng L^−1^ for 8:2 FTUCA. The results obtained for the six compounds included in the certificate of analysis of the CRM (perfluorobutanesulfonic acid, PFHxS, L‐PFOS, perfluoropentanoic acid [PFPeA], PFHxA, and perfluoroheptanoic acid) showed a relative deviation from the certified values of less than 30% (Table S2).

To compare our method with the method of the accredited laboratory, five TWs were sampled three times following the three different approaches: (1) developed method used in this study (polypropylene bottle, ammonium acetate) and analyzed by our laboratory; (2) collection in polypropylene bottles from the accredited laboratory, no ammonium acetate added, analyzed by our laboratory; (3) collection in polypropylene bottles from the accredited laboratory, no ammonium acetate added, analyzed by the accredited laboratory (33 PFAS in common with our study). Detailed results can be found in Tables S3 and S4. Briefly, the comparison of methods 2 and 3 showed that 93% of the results obtained from method 2 were compliant with those of method 3 (relative error of less than 30% of the certified values), including 72% of compliance for the quantified results and 98% of compliance for non‐quantified results (see Table S3). This showed that both analytical methods mostly gave the same results. When comparing methods 1 and 2, 97% of the results were in agreement, including an 85% and 98% agreement for quantified and non‐quantified results, respectively (see Table S4). This shows that results were similar no matter the container or the addition of ammonium acetate. Finally, methods 1 and 3 were also compared, showing that 93% of our results were compliant with those of the accredited laboratory, including 72% and 98% of compliance for quantified and non‐quantified results respectively, assessing the precision of our method, no matter the collection and storage procedure or the analytical method (see Table S3). Differences in concentration were mostly obtained for PFAS that were present at high levels in the blanks, due to lab contamination. Indeed, despite the precautions taken, contamination by a few PFAS was observed in the solvent blanks and field blanks.

The use of a sensitive device to lower the limits of quantifications can explain the more visible contamination. PFBA (at low levels but above LQ) and 6:2 FTS (at very high levels) were detected in the solvent blanks, suggesting contamination from the solvent themselves or from the laboratory during preparation. Most of the field blanks also contained some PFAS, primarily 6:2 FTS, PFBA, perfluoropentane sulfonic acid, PFHxA, PFOA, and L‐PFOS, with mean concentrations of 49.3, 1.80, 1.39, 0.357, 0.350, and 0.166 ng L^−1^, respectively. As contamination levels were very similar between field blanks, the sampling site was ruled out as a source of contamination, and sample preparation was found to be the most likely cause of contamination, either from the laboratory environment or the chemicals added (ammonium acetate, MeOH, FoA). We therefore calculated the blank percentage as described in the QA/QC section and replaced the values by DET or NE when necessary. Although these thresholds allowed us to deal with the unpredictable background PFAS contamination, which was more noticeable due to the high sensitivity of the device, we acknowledge that this approach has limitations and that contamination should be either minimized or addressed through stricter decision thresholds. Finally, open‐source software QGIS was used to map sampling locations and the spatial distribution of PFAS.

### 3.2 PFAS occurrence and levels in drinking waters

**FIGURE 2:**
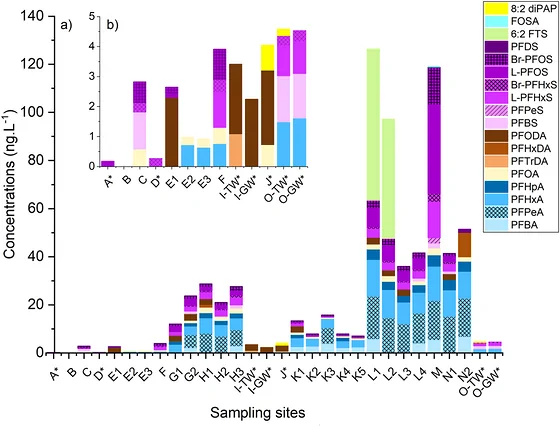
Comparison of total per‐ and polyfluoroalkyl substances (PFAS) concentrations (ng L^−1^) (a) between the 30 drinking water samples and (b) for smaller concentrations. * indicates drinking water supplied by the private catchments. For the location of sampling sites and abbreviations, see legend of the Figure 1.

The sampling campaign comprised 28 TW samples supplied by the CGWT and the Durance River, with most originating from collective water catchments and two groundwater. Frequency of quantification; frequency of detection; and minimum, maximum, median, and mean concentrations of PFAS in the samples are summarized in Table 2. Compounds were considered as detected but not quantifiable when the signal‐to‐noise ratio was between 3 and 10. Only quantified values were included in the calculations of the mean and median. Detailed concentrations of each PFAS congener in each sampling site are provided in Table S5, and Figure 2a,b graphically shows the sum of PFAS for each sampling site.

**TABLE 2:** Summary of per‐ and polyfluoroalkyl substances (PFAS) occurrence in the 30 drinking waters.

PFAS name	MQLs (ng L^−1^)	Frequency of quantification (*n* = 30)	Frequency of detection (*n* = 30)	Minimum (ng L^−1^)	Maximum (ng L^−1^)	Mean (ng L^−1^)	Median (ng L^−1^)
**Perfluoroalkyl carboxylic acids (PFCAs)**							
PFBA	1.10	37%	70%	2.12	6.74	3.72 (*n* = 11)	2.85
PFPeA	5.41	40%	57%	5.45	17.7	11.5 (*n* = 12)	12.3
PFHxA	0.218	73%	90%	0.637	15.4	5.77 (*n* = 22)	3.85
PFHpA	1.07	47%	50%	1.38	4.71	2.75 (*n* = 14)	2.48
PFOA	0.216	63%	87%	0.268	2.60	1.22 (*n* = 19)	0.966
PFNA	1.08	0%	0%	<MQL	<MQL	<MQL	<MQL
PFDA	1.08	0%	27%	<MQL	<MQL	<MQL	<MQL
PFUnDA	0.218	0%	60%	<MQL	<MQL	<MQL	<MQL
PFDoDA	1.09	0%	60%	<MQL	<MQL	<MQL	<MQL
PFTrDA	1.08	3%	97%	1.08	1.08	1.08 (*n* = 1)	1.08
PFTeDA	1.09	0%	73%	<MQL	<MQL	<MQL	<MQL
PFHxDA	1.08	7%	50%	1.17	10.2	5.66 (*n* = 2)	5.66
PFODA	2.17	40%	57%	2.22	2.73	2.42 (*n* = 12)	2.37
**Perfluoroalkyl sulfonic acids (PFSAs)**							
PFBS	1.10	27%	83%	1.23	2.14	1.50 (*n* = 8)	1.45
PFPeS	1.08	3%	40%	2.42	2.42	2.42 (*n* = 1)	2.42
L‐PFHxS	0.860	50%	83%	0.964	14.9	3.05 (n = 15)	2.65
Br‐PFHxS	0.229	60%	73%	0.256	3.46	0.714 (*n* = 18)	0.571
PFHpS	1.09	0%	60%	<MQL	<MQL	<MQL	<MQL
L‐PFOS	0.130	60%	87%	0.145	36.9	4.11 (*n* = 18)	1.68
Br‐PFOS	0.081	63%	80%	0.205	15.5	1.99 (*n* = 19)	1.24
PFNS	1.08	0%	7%	<MQL	<MQL	<MQL	<MQL
PFDS	1.08	3%	50%	1.28	1.28	1.28 (*n* = 1)	1.28
PFUnDS	1.02	0%	3%	<MQL	<MQL	<MQL	<MQL
PFDoDS	0.200	0%	0%	<MQL	<MQL	<MQL	<MQL
PFTrDS	1.03	0%	10%	<MQL	<MQL	<MQL	<MQL
**Perfluoroalkyl ether carboxylic acids (PFECAs)**							
HFPO‐DA	1.09	0%	3%	<MQL	<MQL	<MQL	<MQL
DONA	0.218	0%	100%	<MQL	<MQL	<MQL	<MQL
**Perfluoroalkyl ether sulfonic acid (PFESA)**							
6:2 Cl‐PFESA	0.211	0%	43%	<MQL	<MQL	<MQL	<MQL
**Fluorotelomer sulfonic acids (FTS)**							
6:2 FTS	0.172	7%	10%	49.54	62.8	56.2 (*n* = 2)	56.2
8:2 FTS	1.09	0%	0%	<MQL	<MQL	<MQL	<MQL
**Perfluoroalkane sulfonamides (FASAs)**							
FOSA	0.218	3%	10%	0.512	0.512	0.512 (*n* = 1)	0.512
N‐EtFOSA	1.10	0%	0%	<MQL	<MQL	<MQL	<MQL
N‐MeFOSA	1.08	0%	0%	<MQL	<MQL	<MQL	<MQL
**Perfluoroalkane sulfonamidoacetic acids (FASAAs)**							
L‐N‐EtFOSAA	0.895	0%	0%	<MQL	<MQL	<MQL	<MQL
Br‐N‐EtFOSAA	0.98	0%	0%	<MQL	<MQL	<MQL	<MQL
N‐MeFOSAA	0.216	0%	97%	<MQL	<MQL	<MQL	<MQL
**Polyfluoroalkyl phosphoric acid diester (diPAP)**							
8:2 diPAP	0.216	7%	53%	0.222	0.840	0.531 (*n* = 2)	0.531
**Fluorotelomer unsaturated carboxylic acid (FTUCA)**							
8:2 FTUCA	5.44	0%	0%	<MQL	<MQL	<MQL	<MQL

*Note*: Compounds are arranged in the table by family and by increasing number of carbon atoms.

Abbreviation: DONA, 4.8‐dioxa‐3H‐perfluorononanoic acid; FOSA, perfluorooctane sulfonamide; HFPO‐DA, tetrafluoro‐2 (heptafluoropropoxy)propanoate; MQL, method quantification limit; N‐EtFOSA, N‐ethyl perfluorooctane sulfonamide; N‐EtFOSAA, N‐ethyl perfluorooctane sulfonamido acetic acid; N‐MeFOSA, N‐methyl perfluorooctane sulfonamide; N‐MeFOSAA, N‐methyl perfluorooctane sulfonamido acetic acid; PFBA, perfluorobutanoic acid; PFBS, perfluorobutanesulfonic acid; PFDA, perfluorodecanoic acid; PFDS, perfluorodecane sulfonic acid; PFDoDA, perfluorododecanoic acid; PFDoDS, perfluorododecane sulfonic acid; PFHpA, perfluoroheptanoic acid; PFHpS, perfluoroheptane sulfonic acid; PFHxA, PFHxDA, perfluorodecahexanoic acid; perfluorohexanoic acid; PFHxS, perfluorooctane hexanoic acid; PFNA, perfluorononanoic acid; PFNS, perfluorononane sulfonic acid; PFOA, perfluorooctane carboxylic acid; PFODA, perfluorooctadecanoic acid; PFOS, perfluorooctane sulfonic acid; PFPeA, perfluoropentanoic acid; PFPeS, perfluoropentane sulfonic acid; PFTrDA, perfluorotridecanoic acid; PFTrDS, perfluorotridecane sulfonic acid; PFTeDA, perfluorotetradecanoic acid; PUnDA, perfluoroundecanoic acid; PFUnDS, perfluoroundecane sulfonic acid.

All samples contained detectable levels of some PFAS and 97% of the samples were quantified (at least one PFAS). Among the 38 PFAS investigated, 12 compounds were detected but not quantified (Table 2), 18 were quantified, and eight showed no detectable signal.

The frequency of quantification of each PFAS went from 0% to 73% of the samples (Table 2), and total PFAS concentrations ranged from <0.081 to 126 ng L^−1^ (Figure 2). The highest contamination (126 ng L^−1^) was obtained for site L1, with the highest contribution from PFOS. For other sampling sites, the sums of total PFAS ranged between <0.081 and 119 ng L^−1^, which is in accordance with other works on European drinking water (76–156 ng L^−1^ in France [Boiteux et al., 2012], 34–180 ng L^−1^ in Barcelona [Cserbik et al., 2023]).

## 4 DISCUSSION

### 4.1 PFAS geographical distribution: Differences between water catchments

**FIGURE 3:**
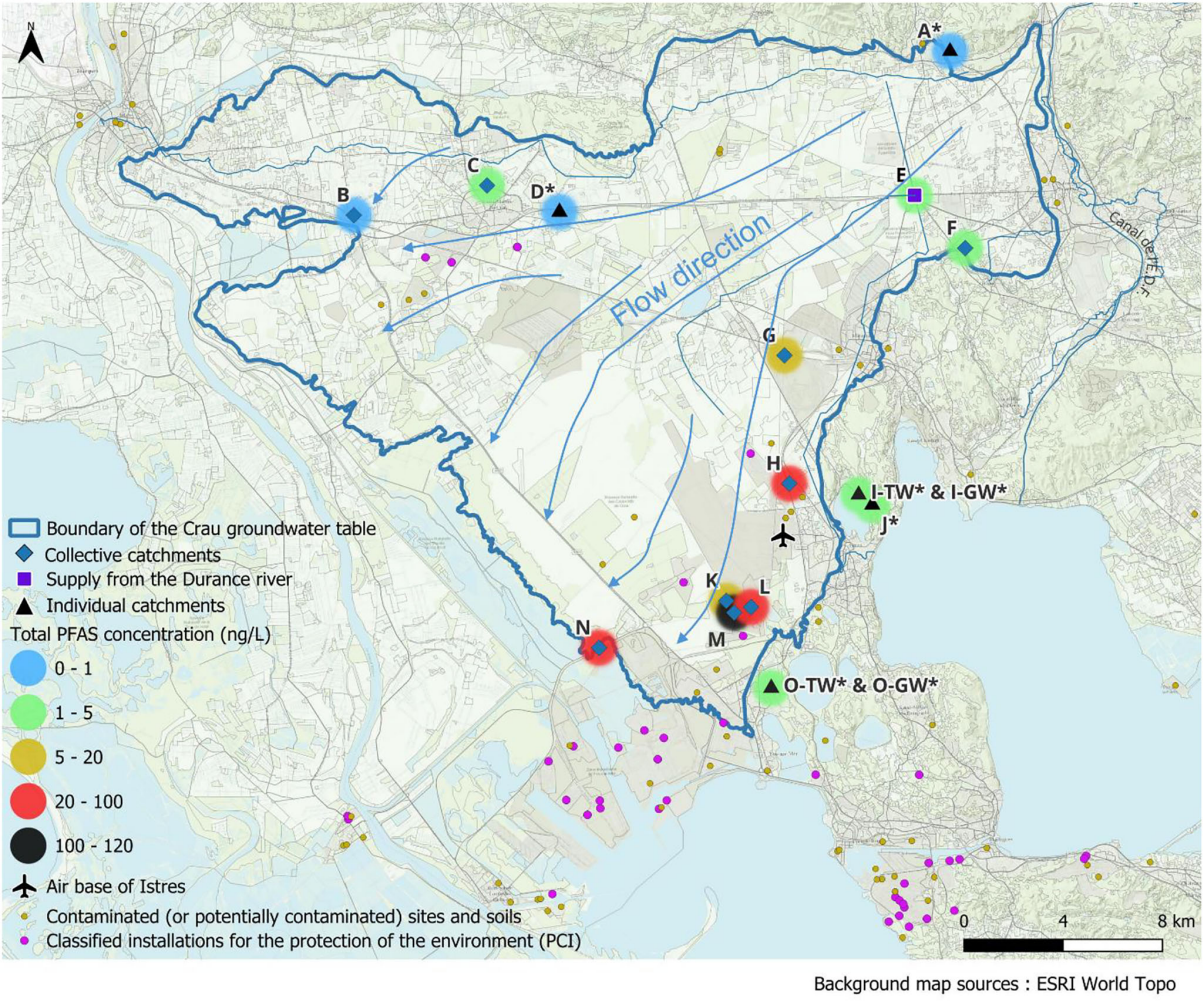
Heat map of the total per‐ and polyfluoroalkyl substances (PFAS) concentration (ng L^−1^) illustrating the spatial distribution of PFAS on the Crau groundwater table (CGWT). * indicates drinking water supplied by the private catchments. For the location of sampling sites and abbreviation, see legend of Figure 1. Groundwater flow directions were obtained from the SYMCRAU.

To assess the influence of geographic location and surrounding environment, the results obtained from individual sampling sites (taps) were aggregated according to their respective water catchments (Figure S1) and were represented in the form of a heat map (Figure 3). This approach was adopted because certain sampled taps may be situated at considerable distances from their catchment (Figure 1).

Water catchments with the highest contamination were G, H, K, L, M and N, with total PFAS concentration ranging between 36–126 ng L^−1^ for L, 119 ng L^−1^ for M, 41.4–51.4 ng L^−1^ for N, 21.0–27.6 ng L^−1^ for H, 12.0–23.8 ng L^−1^ for G, and 7.07–13.3 ng L^−1^ for K.

The high contamination of the drinking water supplied by the water catchment M (∑20 PFAS = 119 ng L^−1^) was also reported by another study. For samples collected in November 2024, ∑20 PFAS ranged from 365 to 750 ng L^−1^ (*n* = 10) (ARS, 2025b). The lower concentration obtained in our study can be explained by the collection of the sample in June, that is, the high‐water season, during which the higher agricultural irrigation may lead to dilution of the contamination, while in the other study, samples were collected in November, during the low‐water season.

The contamination levels quantified in drinking water from catchment L (located in Fos‐sur‐Mer) ranged between 36 and 126 ng L^−1^ (Figure 2) and are consistent with another study that measured contamination between 47 ng L^−1^ and 167 ng L^−1^ between January and May 2025 (IGN Ma Carte, 2025), with the highest concentrations obtained for samples collected in January, February, and March.

Water catchments G, H, K, L, M, and N are located in the middle or downstream of the CGWT (Figure 3), and are surrounded by the major industrial area of Fos and downstream of a military airport. Thus, the differences in contamination levels and profiles over the 28 sites seem to indicate a possible influence of the military airport, firefighting training areas and several industries that are located on the water table since effluents, landfills, and air emission have shown to contaminate soils, rivers, and groundwaters (Alexander et al., 2025). However, the contribution of the industries of the industrial harbor of Fos‐sur‐Mer is less likely to occur since they are located outside and downstream the CGWT. Contamination upstream of the military airport was also observed (catchment H). In addition, PFAS were quantified in the effluents of some of the PCI located in the area (IGN Ma Carte, 2025). Consistently, TWs supplied from water catchments located upstream of the CGWT and far from the industrial area were less contaminated (Figure 3), with the sum of total PFAS ranging from <LQ to 5.39 ng L‐^1^ (water catchments B, C, D*, F). The level of PFAS contamination upstream of the CGWT was comparable to that in the Durance River (catchment E) and in the groundwater at the northern edge of the CGWT (catchment A*). However, the level of contamination downstream of the CGWT was higher than for individual catchments I*, J*, and O* located at the southern edge of the CGWT. Notably, a clear difference was observed between neighboring catchments I*, J*, and H: while the individual catchments I* and J* showed relatively low PFAS concentrations (sum of total PFAS ranging from 2.25 to 3.42 ng L^−^^1^), the collective catchment H displayed markedly higher values (21.0–28.7 ng·L^−^^1^). The higher contamination measured downstream the CGWT might be due to the accumulation of contaminants along the water table, coming from industrial discharge of industries located on the CGWT. However, the activities from the industrial harbor of Fos‐sur‐Mer, cannot be responsible for the water table contamination as they are located downstream and outside of the CGWT. Alternatively, it could be due to differences in catchment installation, such as the depth, nature of the pipes, the difference in filters/treatments used by the different collective treatment stations and the individual catchment treatments or the lithological profile of the soil, which can influence the transport and leaching of PFAS. Nevertheless, the transport of volatile PFAS precursors can also contribute to PFAS contamination, as they can be transformed into stable PFAS, mainly PFCAs and PFSAs, through biodegradation (Zhang et al., 2022), atmospheric oxidation (Ellis et al., 2004), wastewater treatment plants, drinking water treatment processes, particularly chlorination‐driven transformation (Sun et al., 2025), and advanced oxidation processes (Anumol et al., 2016). Indeed, the transformation of fluorotelomers can occur with time in groundwater (Alexander et al., 2025), which can explain the higher contamination downstream of the aquifer.

### 4.2 PFAS profiles and chemical signatures

Eleven of the 18 quantified compounds in water were short‐chain PFCAs and PFSAs, with a high frequency of quantification (Table 2). PFTrDA, N‐MeFOSAA, and PFHxA were detected in the highest number or samples (90%) but PFTrDA and N‐MeFOSAA were quantified in only one sample and zero samples, respectively. PFHxA was the most frequently quantified compound (73%), followed by PFOA and Br‐PFOS (63%), Br‐PFHxS and L‐PFOS (60%), and L‐PFHxS (50%). Nevertheless, this should be interpretated with caution as the MQL vary among PFAS congeners. Strong occurrence of short‐chain PFCAs and PFSAs in drinking water has already been reported by previous studies in France (Boiteux et al., 2012; Cserbik et al., 2023; Kaboré et al., 2018; Schwanz et al., 2016; Teymoorian et al., 2025) and worldwide (Teymoorian et al., 2023). In Canadian TWs, PFBA, PFOS, PFHxA, and PFOA—representative short‐chain PFAS—dominated the overall PFAS profile, with concentrations ranging from 3.5 to 4.9 ng L^−1^ (Kaboré et al., 2018). However, long‐chain PFAS were also frequently detected, with PFNA and PFDA detected in at least 84% of the samples (*n* = 19) (Kaboré et al., 2018). In the present study, PFNA was not detected, and PFDA was detected (but not quantified) in only 27% of the samples. Concerning other long‐chain PFAS, C_11_‐ to C_18_‐PFCAs were detected and quantified in 50%–97% and 0%–40% of the samples respectively, and C_9_–C_13_ PFSAs were detected and quantified in 0%–50% and 0%–3% of the samples, respectively. Perfluorooctane sulfonamide was also found in 53% of the samples (Kaboré et al., 2018), while in only one sample (3%) here.

Short‐chain PFAS also displayed the highest concentrations, with ranges between 0.637–15.4 ng L^−1^ for PFHxA, 0.145–36.9 ng L^−1^ for L‐PFOS, 0.205–15.5 ng L^−1^ for Br‐PFOS, and 0.964–14.9 for L‐PFHxS. PFPeA and PFBA, which were quantified in 40% and 37% of the samples respectively, also displayed high concentrations: 5.45–17.7 ng L^−1^ for PFPeA, and 2.12–6.74 ng L^−1^ for PFBA (Table 2). This pattern was likewise documented in the southern Lyon area, France (Teymoorian et al., 2025). The higher quantification of short‐chain PFAS is consistent with their higher solubility compared to long‐chain PFAS, due to their lower hydrophobicity, stronger polarity, and therefore the lower adsorption potential to soil leading to a leaching from soils to groundwater (Gellrich et al., 2012). It is also due to the increasing replacement of long‐chain PFAS by shorter chain PFAS (Wang et al., 2013) or the obtention of short chain PFAS by degradation of longer chain precursors in the environment and biota (Ellis et al., 2004; Zhang et al., 2022).

Linear and branched isomers of PFHxS, PFOS, and N‐EtFOSAA were analyzed separately and integrated separately as the chromatographic resolution was sufficient. In fact, the study of chemical signature or profile can help to identify emission sources, since topomerization yields only the linear form while electrofluorination yields a presence of a mixture of linear and branched isomers (3M, 1999; Benskin et al., 2010), and since the manufacturing process can be specific to certain industrial sectors.

The coexistence of two manufacturing processes, their temporal variability in use, and the limited number of studies addressing individual isomers hinder a comprehensive assessment of isomeric ratios and their environmental fate. Nevertheless, a review highlighted that isomers exhibit distinct environmental behaviors, with differential adsorption depending on the matrix considered (Schulz et al., 2020).

In the present study, for the 15 samples where both isomers were quantified, a single pattern of Br‐PFHxS contribution was obtained and ranged between 23% and 34%, with a mean of 28%. For branched over linear PFOS ratios, three different patterns were observed: they went from 86% to 91% for water catchment H (*n* = 3); from 40% to 54% for water catchments G (*n* = 2), L (*n* = 3), M and for sample N1; and from 131% to 165% for water catchment K (*n* = 5). PFOS isomer ratios were relatively consistent within each water catchment. However, no clear relationship with location or with a specific emission source was observed. Different isomer ratios were found in two neighboring catchments separated by only about 2.3 km. Near the Istres military air base, branched PFOS represented 46%–48% of total PFOS, while linear PFOS accounted for 52%–54%. This differs from historical PFOS‐containing AFFF formulations, in which linear PFOS generally represented 70%–80% of total PFOS (3M, 1999; ITRC, 2026). Since recent AFFF no longer contain PFOS, and telomer‐based AFFF contain only linear compounds, the high proportion of branched PFOS is unlikely to result from direct PFAS emissions. It may instead reflect differences in environmental behavior between isomers. In particular, the preferential adsorption of linear PFOS onto soils and sediments, as reported by Schulz et al. (2020), could explain the relative enrichment of branched PFOS in groundwater and, consequently, in drinking water. The variation in PFOS isomer ratios may therefore be explained by either three contamination sources, differential sorption onto sediments or specific degradation of precursors, all of which may be emphasized by the difference in sample location and/or the time of the potential point contamination that can lead to the alteration of the initial isomer concentrations.

### 4.3 Influence of water treatments on PFAS removal from TW

The presence of PFAS in drinking water indicated, as expected, the inefficiency of conventional water treatment such as chlorination and ozone in removing PFAS. In addition, in order to assess the impact of private filters and treatment systems on PFAS levels and profiles, drinking water produced from a private well, treated with domestic treatments (‐TW), was compared with untreated water from the well (raw groundwater [‐GW]). At site I*, the groundwater (I‐GW*) underwent treatment involving a 10 µm filter, a softener, reverse osmosis with activated carbon, and UV treatment. At site O*, the groundwater (O‐GW*) was filtered using a cotton cartridge, a carbon filter, a glyphosate filter, and a taste filter. For both sites, no significant changes in concentration were observed between the treated and the GW, and the profiles of compounds quantified were globally the same, although the low concentrations obtained limited the evaluation of the device's effectiveness.

This can be due to the ineffectiveness of the treatments or to the desorption from saturated filters and subsequent release of PFAS into the treated water due to excessive use. Indeed, most common treatments such as coagulation/sedimentation, sand filtration, chlorination and ozonation were found to be ineffective (Boiteux et al., 2017) or only effective for long chain PFAS, although ozonation can induce transformation of precursors (Boiteux et al., 2017).

Adsorption on activated carbon (Belkouteb et al., 2020; Li et al., 2020) and anion exchange (Li et al., 2020) were found to be more effective for long chain PFAS, with a better performance for PFSAs than PFCAs (Belkouteb et al., 2020). The efficiency of adsorption processes therefore depends on the nature and hydrophobicity of the PFAS but also on the sample pH, the presence of dissolved organic matter (Li et al., 2020) on the duration of use of the system and the operating flow rate (Belkouteb et al., 2020), requiring frequent filter regeneration to prevent the release of pollutants. Nevertheless, reverse osmosis and nanofiltration (Boiteux et al., 2017) have been found to be effective for long and short chain PFAS, though being very expensive. However, assessments of treatment performance are usually done for a concise list of PFAS, and information about precursors treatment in drinking water is scarce. Domestic reverse osmosis systems also achieved removal rates exceeding 90% (Cserbik et al., 2023). In the present study, however, the presence of a reverse osmosis system at site I* did not reduce PFAS contamination, a result likely attributable to excessive filter use.

## 5 RISK ASSESSMENT AND COMPARISON TO THE 2020/2184 EUROPEAN DWD

To assess the potential health risks associated with drinking water consumption, the results were compared to the EU DWD 2020/2184 guidelines of 100 ng L^−1^ for ∑20 PFAS and 500 ng L^−1^ for total PFAS, to a French guidance value of 20 ng L^−1^ for ∑4 PFAS (PFOA, PFOS, PFNA, and PFHxS) and to the TWI value of 4.4 ng kg_bw_^−1^ for ∑4 PFAS (PFOA, PFOS, PFNA, and PFHxS). For all the calculations, the concentrations of the linear and branched isomers were added together. The total PFAS concentration was below the DWD limit for all samples (concentrations from 0 to 126 ng L^−1^). Ultra‐short chain PFAS were not investigated in the present work and might have increased the sum of total PFAS. The 20 PFAS regulated under the EU DWD 2020/2184 were investigated and for the 11 quantified compounds, the sum did not exceed the limit value (0–60.8 ng L^−1^) except pour 1 sample (119 ng L^−1^, site M). The same sample also exceeded the French guidance value of 20 ng L^−1^ for the sum of four PFAS (PFOA, PFOS, PFNA, and PFHxS) (HCSP, 2024), reaching 73.43 ng L^−1^. By comparison, in the heavily impacted area of southern Lyon, France—where two PFAS manufacturing facilities (Daikin and Arkema) are located—∑20 PFAS concentrations in TW ranged from 40 to 200 ng L^−1^ (ARS, 2025a). The discrepancy with the present study is most likely attributable to the absence of PFAS production sites in the investigated area.

Food, especially fish, eggs, fruits, and vegetables (EFSA CONTAM Panel et al., 2020), and water consumption, both TW and bottled water, can highly contribute to human exposure. Indeed, PFAS have also been found in bottled water, with a total PFAS concentration of 116 ng L^−1^ (Schwanz et al., 2016). To determine the health risk assessment, estimated weekly intake (EWI) values were calculated for adults and children and compared to the TWI of 4.4 ng kg_bw_^−1^ established by the EFSA (EFSA CONTAM Panel et al., 2020) for the sum of four PFAS (PFOA, PFOS, PFNA, and PFHxS). To do so, an average adult bw of 60 kg (25 kg for children), a daily drinking water intake of 2 L (EFSA CONTAM Panel et al., 2020), and an assumed 20% contribution of water to total PFAS exposure (World Health Organization, 2022) were used in the calculation: EWI (ng kg_bw_^−1^) = (concentration (ng L^−1^) × 2 L × 7 days)/(60 kg × 20%). EWI values of all samples can be found in Table S6. Under these assumptions, regular consumption of the water from 11 sites would result in exceeding the TWI and lead to a potentially substantiated health risk. This represent 39% of the drinking water samples (*n* = 28). Concerning children, the TWI is exceeded by 17 samples (61% of the samples). Expanding risk assessment to incorporate a broader suite of PFAS, including precursors, would improve the robustness of exposure and health risk evaluations.

## 6 CONCLUSIONS

An LC–MS/MS method was developed to quantify 38 PFAS in drinking water collected from 28 tap‐ and two ground‐water sources within a multi‐activity industrial region. PFAS were detected in 97% of samples, with 18 compounds quantified. Detected profiles and concentrations were consistent with previous regional studies, with PFHxA, PFOS, PFHxS, and PFOA among the most frequently observed compounds. Spatial analysis indicated higher contamination levels downstream of the water table compared to upstream locations.

Only one sample exceeded the limits of European DWD (EU 2020/2184) (Σ20 PFAS) and of the French guidance (Σ4 PFAS). However, EWIs based on regular TW consumption exceeded EFSA tolerable intake values at 12 sites for adults and 18 sites for children, indicating potential public health concerns. Despite including the 20 PFAS regulated under the European DWD, the set of 38 PFAS analyzed did not cover ultra‐short‐chain compounds, such as trifluoroacetic acid. This represents a limitation of the study, as these compounds have been shown to significantly contribute to contamination across environmental matrices, including drinking water.

The study highlights the need for further investigation of PFAS sources through isomer profiling and assessment of precursor compounds using total oxidizable precursor assays or suspect screening. Additional research is required to improve treatment technologies, particularly for short‐chain PFAS and precursors. Overall, this work provides high‐resolution mapping of PFAS contamination in France's largest industrial area and supports future comparative assessments between PFAS‐use and PFAS‐production sites to inform exposure, risk assessment, and regulatory decision‐making.

## AUTHOR CONTRIBUTIONS

**M. Dijoux**: Methodology; resources; writing—original draft. **A. Piram**: Funding acquisition; supervision; validation; writing—review and editing. **S. Augy**: Conceptualization; funding acquisition; supervision; validation; writing—review and editing. **A. Austruy**: Conceptualization; funding acquisition; supervision; validation; writing—review and editing. **A. Souloumiac**: Investigation; resources. **L. De Jong**: Writing—review and editing. **E. Gonzales‐Camoin**: Investigation; resources. **J. Dron**: Conceptualization; funding acquisition; supervision; validation; writing—review and editing. **P. Chamaret**: Conceptualization; writing—review and editing. **X. Moreau**: Funding acquisition; writing—review and editing. **S. Gori**: Conceptualization; funding acquisition; project administration. **P. Wong‐Wah‐Chung**: Writing—review and editing. **P. Doumenq**: Conceptualization; funding acquisition; investigation; project administration; supervision; writing—review and editing.

## CONFLICT OF INTEREST STATEMENT

The authors declare no conflicts of interest.

## Supporting information



The supplementary file contains the list of target PFAS, the individual concentrations of PFAS for the 30 water samples, the results of the CRM analysis using the developed method, and two tables showing the comparison between the developed method and the method of the accredited laboratory to support the information given in section 3.1. *Method Performance Parameters and data Analysis*.
