# Supplementary material for: Drinking waters contamination by per‐ and poly‐fluoroalkyl substances of the anthropized area of Fos‐Berre, France
Source: J Environ Qual. 2026 Sep 21;55(5):e70241. doi: 10.1002/jeq2.70241 (PMC13594370; doi:10.1002/jeq2.70241)
Supplement: Supplementary file 1 [file JEQ2-55-0-s001.docx]

Supplementary information

PFAS investigations in drinking waters of the highly anthropized area of Fos-Berre, France

M. Dijoux^a, b^, A. Piram^b*^, S. Augy^a^, A. Austruy^c^, A. Souloumiac^c^, L. De Jong^d^, E. Gonzales‑Camoin^c^, J. Dron^c^, P. Chamaret^c^, X. Moreau^d^, S. Gori^a^, P. Wong‑Wah-Chung^b^, P. Doumenq^b^

^a^ Société ABO-ERG Environnement, Vitrolles, France

^b^ Aix Marseille Univ, LCE, Marseille, France

^c^ Institut Écocitoyen pour la Connaissance des Pollutions, Fos-sur-Mer, France

^d^ Aix Marseille Univ, Avignon Univ, CNRS, IRD, IMBE, Marseille, France

[Table S1. Information about the target compounds. Compounds are arranged in the table by family and by increasing number of carbon atoms.*No information given for the consideration of isomers as a group or as individuals. MQL = Method quantification limit. Acids correspond to the acidic form of the purchased salt.	2](#_Toc233123273)

[Table S2. Comparison of the results obtained from the analysis of a CRM using the developed method with those from the certificate of analysis. Relative deviations were calculated as (experimental value)/(certified value)*100.	4](#_Toc233123274)

[Table S3. Comparison of the results between our study and the accredited laboratory, following three different methods: 1) Developed method used in this study (polypropylene bottle, ammonium acetate) and analysed by our laboratory, 2) collection in polypropylene bottle from the accredited laboratory, no ammonium acetate added, analysed by our laboratory, 3) collection in polypropylene bottle from the accredited laboratory, no ammonium acetate added, analysed by the accredited laboratory. For the results from the accredited laboratory, all compounds were analysed with the method PR EN 17892 except for PFHxDA, PFODA, N-MeFOSAA, 8:2 diPAP that were analysed with an internal method. Relative deviations were calculated as (experimental value)/(accredited value)*100.	5](#_Toc233123275)

[Table S4. Comparison of methods 1 and 2: 1) Developed method used in this study (polypropylene bottle, ammonium acetate) and analysed by our laboratory, 2) collection in polypropylene bottle from the accredited laboratory, no ammonium acetate added, analysed by our laboratory. Relative deviations were calculated as (Method1)/(Method 2)*100.	7](#_Toc233123276)

[Table S5. Individual PFAS concentration (ng.L^-1^) for the 30 samples, DET = detected (when blank percentage between 25% and 50% of the signal), < LQ = below the limit of quantification, ND = non detected, NE = non exploitable (when blank percentage > 50% of the signal).	9](#_Toc233123277)

[Table S6. Estimated Weekly Intake (EWI) values (ng.kg_bw_ ^-1^) for adults and children using an average adult body weight of 60 kg (25 kg for children), a daily drinking water intake of 2 L and an assumed 20% contribution of water to total PFAS exposure. Samples exceeding the TWI of 4,4 ng.kg_bw_ ^-1^ are highlighted in orange.	11](#_Toc233123278)

[Figure S1. Comparison of average total PFAS concentrations (ng.L-1) between water catchments. A to O: Catchment identity. * Indicate drinking water supplied by the private catchments. For the location of sampling sites, see legend of Figure 1.	11](#_Toc216876830)

Table S1. Information about the target compounds. Compounds are arranged in the table by family and by increasing number of carbon atoms.*No information given for the consideration of isomers as a group or as individuals. MQL = Method quantification limit. Acids correspond to the acidic form of the purchased salt.

| **Compound name** | **Acronym** | **Included in ISO 21675*** | **Included in EU DWD*** | **Included in PCI regulation*** | **CAS** | **Quantitative transition (Q1)** | **Qualitative transition (Q2)** | **Ion ratio (Q1/Q2)** | **Internal standard** | **MQL (ng.L^‑1^)** |
| --- | --- | --- | --- | --- | --- | --- | --- | --- | --- | --- |
| ***Perfluoroalkyl carboxylic acids (PFCAs)*** | | | | | | | | | | |
| Perfluoro-n-butanoic acid | PFBA | X | X | X | 375-22-4 | 212.9 → 169 | 212.9 → 19 | 0.002 | ^13^C_6_-PFHxA | 1.1 |
| Perfluoro-n-pentanoic acid | PFPeA | X | X | X | 2706-90-3 | 263 → 219 | 263 → 69 | 0.01 | ^13^C_6_-PFHxA | 5.41 |
| Perfluorohexanoic acid | PFHxA | X | X | X | 307-24-4 | 313 → 269 | 313 → 119 | 0.07 | ^13^C_6_-PFHxA | 0.218 |
| Perfluoroheptanoic acid | PFHpA | X | X | X | 375-85-9 | 362.9 → 318.8 | 362.9 → 168.9 | 0.34 | ^13^C_6_-PFHxA | 1.07 |
| Perfluorooctanoic acid | PFOA | X | X | X | 335-67-1 | 413 → 169 | 413 → 219 | 0.54 | ^13^C_8_-PFOA | 0.216 |
| Perfluorononanoic acid | PFNA | X | X | X | 375-95-1 | 463 → 419 | 463 → 219 | 0.36 | ^13^C_9_-PFDA | 1.08 |
| Perfluorodecanoic acid | PFDA | X | X | X | 335-76-2 | 513 → 468.9 | 513 → 218.9 | 0.20 | ^13^C_9_-PFDA | 1.08 |
| Perfluoroundecanoic acid | PFUnDA | X | X | X | 2058-94-8 | 563 → 268.9 | 563 → 319 | 0.70 | ^13^C_9_-PFDA | 0.218 |
| Perfluorododecanoic acid | PFDoDA | X | X | X | 307-55-1 | 613 → 318.9 | 613 → 169 | 0.98 | ^13^C_9_-PFDA | 1.09 |
| Perfluorotridecanoic acid | PFTrDA | X | X | X | 72629-94-8 | 663 → 318.9 | 663 → 219 | 0.94 | ^13^C_9_-PFDA | 1.08 |
| Perfluorotetradecanoic acid | PFTeDA | X |  | X | 376-06-7 | 712.9 → 219 | 712.9 → 269 | 0.78 | ^13^C_9_-PFDA | 1.09 |
| Perfluorohexadecanoic acid | PFHxDA | X |  | X | 67905-19-5 | 812.9 → 768.8 | 812.9 → 218.9 | 7.67 | ^13^C_9_-PFDA | 1.08 |
| Perfluorooctadecanoic acid | PFODA | X |  | X | 16517-11-6 | 912.9 → 868.9 | 912.9 → 219 | 0.12 | ^13^C_9_-PFDA | 2.17 |
| ***Perfluoroalkyl sulfonic acids (PFSAs)*** | | | | | | | | | | |
| Perfluorobutane(-1-)sulfonic acid | PFBS | X | X | X | 375-73-5 | 299 → 79.9 | 299 → 98.9 | 0.38 | ^13^C_8_-PFOS | 1.10 |
| Perfluoro-1-pentanesulfonic acid | PFPeS |  | X | X | 2706-91-4 | 348.7 → 79.9 | 348.7 → 98.9 | 0.38 | ^13^C_8_-PFOS | 1.08 |
| Perfluorohexanesulfonic acid (potassium salt) | Linear PFHxS | X | X | X | 355-46-4 | 399 → 79.9 | 399 → 98.9 | 0.34 | ^13^C_8_-PFOS | 0.860 |
|  | Branched PFHxS |  |  |  |  |  |  | 0.20 | ^13^C_8_-PFOS | 0.229 |
| Perfluoro-1-heptanesulfonic acid | PFHpS | X | X | X | 375-92-8 | 448.82 → 79.9 | 448.82 → 98.9 | 0.32 | ^13^C_8_-PFOS | 1.09 |
| Perfluoro-1-octanesulfonic acid | Linear PFOS | X | X | X | 1763-23-1 | 499 → 79.9 | 499 → 98.9 | 0.30 | ^13^C_8_-PFOS | 0.130 |
|  | Branched PFOS |  |  |  |  |  |  | 0.11 | ^13^C_8_-PFOS | 0.081 |
| Perfluoro-1-nonanesulfonic acid | PFNS |  | X | X | 68259-12-1 | 548.8 → 79.9 | 548.8 → 99 | 0.30 | ^13^C_8_-PFOS | 1.08 |
| Perfluoro-1-decanesulfonic acid | PFDS | X | X | x | 335-77-3 | 599 → 80 | 599 → 98.9 | 0.23 | ^13^C_8_-PFOS | 1.08 |
| Perfluorododecanesulfonic acid | PFDoDS |  | X | X | 79780-39-5 | 698.8 → 79.9 | 698.8 → 98.9 | 0.25 | ^13^C_8_-PFOS | 0.200 |
| Perfluroundecanesulfonic acid | PFUnDS |  | X | X | 749786-16-1 | 649 → 79.9 | 649 → 98.9 | 0.27 | ^13^C_8_-PFOS | 1.02 |
| Perfluorotridecanesulfonic acid | PFTrDS |  | X | X | 791563-89-8 | 749 → 80 | 749 → 99 | 0.38 | ^13^C_8_-PFOS | 1.03 |
| ***Perfluoroalkyl ether carboxylic acids (PFECAs)*** | | | | | | | | | | |
| Tetrafluoro-2-(heptafluoropropoxy)propanoate | HFPO-DA | X |  | X | 13252-13-6 | 284.9 → 168.9 | 284.9 → 185 | 0.69 | ^13^C_3_-HFPO-DA | 1.09 |
| 4.8-dioxa-3H-perfluorononanoic acid | DONA | X |  | X | 919005-14-4 | 376.9 → 250.9 | 376.9 → 84.9 | 0.50 | ^13^C_3_-HFPO-DA | 0.218 |
| ***Perfluoroalkyl ether sulfonic acids (PFESA)*** | | | | | | | | | | |
| 9-chlorohexadecafluoro-3-oxanonane-1-sulfonic acid | 6:2 Cl-PFESA | X |  |  | 73606-19-6 /756426-58-1 | 531 → 351 | 531 → 35 | 0.04 | ^13^C_8_-PFOS | 0.211 |
| ***Fluorotelomer sulfonic acids (FTS)*** | | | | | | | | | | |
| 1H.1H.2H.2H-Perfluorooctane sulfonic acid | 6:2 FTS | X |  |  | 27619-97-2 | 427 → 407 | 427 → 81 | 0.48 | ^13^C_8_-PFOS | 0.172 |
| 1H. 1H. 2H. 2H-Perfluorodecane sulfonic acid | 8:2 FTS | X |  |  | 39108-34-4 | 527 → 81 | 527 → 507 | 2.04 | ^13^C_8_-PFOS | 1.09 |
| ***Perfluoroalkyl sulfonamides (FASAs)*** | | | | | | | | | | |
| Perfluorooctane sulfonamide | FOSA | X |  |  | 754-91-6 | 498 → 78 | 498 → 64 | 0.11 | d_5_-NEtFOSAA | 0.218 |
| Sulfuramid (N-ethyl perfluorooctane sulfonamide) | N-EtFOSA | X |  |  | 4151-50-2 | 526 → 219 | 526 → 169 | 1.13 | d_5_-NEtFOSAA | 1.01 |
| N-methyl perfluorooctane sulfonamide | N-MeFOSA | X |  |  | 31506-32-8 | 512 → 219 | 512 → 169 | 1.09 | d_3_-NMeFOSAA | 1.08 |
| ***Perfluoroalkyl sulfonamidoacetic acids (FASAAs)*** | | | | | | | | | | |
| N-ethyl perfluorooctane sulfonamido acetic acid | Linear N‑EtFOSAA | X |  |  | 2991-50-6 | 584 → 419 | 584 à 483 | 0.52 | d_5_-NEtFOSAA | 0.895 |
|  | Branched N‑EtFOSAA |  |  |  |  |  |  | 0.70 | d_5_-NEtFOSAA | 0.981 |
| N-methyl perfluorooctane sulfonamido acetic acid | N-MeFOSAA | X |  |  | 2355-31-9 | 570 → 419 | 570 → 483 | 0.50 | d_3_-NMeFOSAA | 0.216 |
| ***Polyfluoroalkyl phosphoric acid diester (diPAP)*** | | | | | | | | | | |
| Bis[2-(perfluorooctyl)ethyl] phosphate | 8:2 diPAP | X |  |  | 678-41-1 | 989 → 79 | 989 → 542.9 | 0.76 | d_3_-NMeFOSAA | 0.216 |
| ***Fluorotelomer unsaturated carboxylic acid (FTUCA)*** | | | | | | | | | | |
| 2H-perfluoro-2-decenoic acid | 8:2 FTUCA | X |  |  | 70887-84-2 | 457 → 343 | 457 → 393 | 0.05 | ^13^C_6_-PFHxA | / |
|  |  |  |  |  |  | 457 → 393 → 193 | / | / | ^13^C_6_-PFHxA | 5.44 |

Table S2. Comparison of the results obtained from the analysis of a CRM using the developed method with those from the certificate of analysis. Relative deviations were calculated as (experimental value)/(certified value)*100.

|  | Certified value (ng.L^-1^) | Uncertainty (ng.L^-1^) | Present study (ng.L^-1^) (n=3) | Relative deviation to the certified values (%) |
| --- | --- | --- | --- | --- |
| PFPeA | 4 | 1 | 3.5 | 88% |
| PFHxA | 7.4 | 1 | 5.5 | 74% |
| PFHpA | 3.7 | 0.7 | 3.5 | 95% |
| PFNA (indicative value) | 3.9 | 1.4 | 2.4 | 62% |
| PFBS | 5.5 | 1.4 | 4.9 | 89% |
| PFHxS | 3.6 | 1 | 4.7 | 131% |
| L-PFOS | 9.6 | 1.7 | 11.6 | 121% |

Table S3. Comparison of the results between our study and the accredited laboratory, following three different methods: 1) Developed method used in this study (polypropylene bottle, ammonium acetate) and analysed by our laboratory, 2) collection in polypropylene bottle from the accredited laboratory, no ammonium acetate added, analysed by our laboratory, 3) collection in polypropylene bottle from the accredited laboratory, no ammonium acetate added, analysed by the accredited laboratory. For the results from the accredited laboratory, all compounds were analysed with the method PR EN 17892 except for PFHxDA, PFODA, N-MeFOSAA, 8:2 diPAP that were analysed with an internal method. Relative deviations were calculated as (experimental value)/(accredited value)*100.

|  |  |  | M | | | Field blank of M | | | L4 | | | H3 | | | A* | | |
| --- | --- | --- | --- | --- | --- | --- | --- | --- | --- | --- | --- | --- | --- | --- | --- | --- | --- |
|  | Uncertainty to the LQ | LQ | Accredited results (ng.L^-1^) | Results from this study (ng.L^-1^) (relative deviation (%) to accredited values) | | Accredited results (ng.L^-1^) | Accredited results  (ng.L^-1^) | | Accredited results (ng.L^-1^) | Results from this study (ng.L^-1^) (relative deviation (%) to accredited values) | | Accredited results (ng.L^-1^) | Results from this study (ng.L^-1^) (relative deviation (%) to accredited values) | | Accredited results (ng.L^-1^) | Results from this study (ng.L^-1^) (relative deviation (%) to accredited values) | |
|  |  |  | Method 3 | Method 2 | Method 1 | Method 3 | Method 2 | Method 1 | Method 3 | Method 2 | Method 1 | Method 3 | Method 2 | Method 1 | Method 3 | Method 2 | Method 1 |
| PFBA | 33% | 1 | 61.5 | 5.68 (9 %) | 5.41 (9 %) | <1.01 | <1.1 | <1.1 | 4.71 | 4.71 (100 %) | 4.02 (85 %) | 4.18 | 2.53 (61 %) | 2.85 (68 %) | <1.00 | <1.1 | <1.1 |
| PFPeA | 30% | 1 | 91.8 | 17.36 (19 %) | 16.3 (18 %) | <1.01 | <5.41 | <5.41 | 15.8 | 13.72 (87 %) | 12.47 (79 %) | 8.66 | 6.66 (77 %) | 6.78 (78 %) | <1.00 | <5.41 | <5.41 |
| PFHxA | 30% | 1 | 16.2 | 15.25 (94 %) | 14.33 (89 %) | <1.01 | <0.22 | 0.3 | 8.91 | 10.19 (114 %) | 8.68 (97 %) | 6.5 | 4.66 (72 %) | 4.87 (75 %) | <1.00 | <0.22 | <0.22 |
| PFHpA | 26% | 1 | 5.78 | 4.39 (76 %) | 4.71 (82 %) | <1.01 | <1.07 | <1.07 | 3.07 | 3.54 (115 %) | 3.18 (104 %) | 2.49 | 2.44 (98 %) | 2.09 (84 %) | <1.00 | <1.07 | <1.07 |
| PFOA | 29% | 1 | 3.27 | 2.52 (77 %) | 2.6 (80 %) | <1.01 | <0.22 | <0.22 | 1.82 | 1.79 (98 %) | 1.33 (73 %) | 2.04 | 1.65 (81 %) | 1.78 (87 %) | <1.00 | <0.22 | <0.22 |
| PFNA | 32% | 1 | <1.00 | <1.08 | <1.08 | <1.01 | <1.08 | <1.08 | <1.00 | <1.08 | <1.08 | <1.00 | <1.08 | <1.08 | <1.00 | <1.08 | <1.08 |
| PFDA | 27% | 1 | <1.00 | <1.08 | <1.08 | <1.01 | <1.08 | <1.08 | <1.00 | <1.08 | <1.08 | <1.00 | <1.08 | <1.08 | <1.00 | <1.08 | <1.08 |
| PFUnDA | 19% | 1 | <1.00 | <0.22 | <0.22 | <1.01 | <0.22 | <0.22 | <1.00 | <0.22 | <0.22 | <1.00 | <0.22 | <0.22 | <1.00 | <0.22 | <0.22 |
| PFDoDA | 35% | 1 | <1.00 | <1.09 | <1.09 | <1.01 | <1.09 | <1.09 | <1.00 | <1.09 | <1.09 | <1.00 | <1.09 | <1.09 | <1.00 | <1.09 | <1.09 |
| PFTrDA | 33% | 1 | <1.00 | <1.08 | <1.08 | 1.1 | <1.08 | <1.08 | <1.00 | <1.08 | <1.08 | <1.00 | <1.08 | <1.08 | <1.00 | <1.08 | <1.08 |
| PFTeDA | 32% | 1 | <1.00 | <1.09 | <1.09 | 1.1 | <1.09 | <1.09 | <1.00 | <1.09 | <1.09 | <1.00 | <1.09 | <1.09 | <1.00 | <1.09 | <1.09 |
| PFHxDA | 45% | 1 | <1.00 | <1.08 | <1.08 | <1.01 | <1.08 | <1.08 | <1.00 | <1.08 | <1.08 | <1.00 | <1.08 | <1.08 | <1.00 | <1.08 | <1.08 |
| PFODA | 45% | 1 | <1.00 | NE | NE | <1.01 | 6.6 | 3.4 | <1.00 | NE | NE | <1.00 | NE | NE | <1.00 | NE | NE |
| HFPO-DA | 45% | 5 | <5.00 | <1.09 | <1.09 | <5.05 | <1.09 | <1.09 | <5.00 | <1.09 | <1.09 | <5.00 | <1.09 | <1.09 | <5.00 | <1.09 | <1.09 |
| DONA | 45% | 1 | <1.00 | <0.22 | <0.22 | <1.01 | <0.22 | <0.22 | <1.00 | <0.22 | <0.22 | <1.00 | <0.22 | <0.22 | <1.00 | <0.22 | <0.22 |
| 6:2 FTS | 45% | 1 | 25.6 | NE | NE | 1.09 | 100.7 | 102.7 | 41.7 | NE | NE | 3.42 | NE | NE | 1.35 | NE | NE |
| 8:2 FTS | 45% | 1 | <9.78 | NE | NE | <10.1 | 0.6 | 0.57 | <9.87 | NE | NE | <9.89 | NE | NE | <9.88 | NE | NE |
| 6:2 Cl-PFESA | 45% | 1 | <1.00 | <0.21 | <0.21 | <1.01 | <0.21 | <0.21 | <1.00 | <0.21 | <0.21 | <1.00 | <0.21 | <0.21 | <1.00 | <0.21 | <0.21 |
| PFBS | 36% | 1 | 1.76 | 2.11 (120 %) | 2.14 (122 %) | <1.01 | <1.1 | <1.1 | <1.00 | 1.4 | 1.4 | 1.2 | 1.57 (131 %) | 1.56 (130 %) | <1.00 | <1.1 | <1.1 |
| PFPeS | 32% | 1 | 2.06 | 2.36 (115 %) | 2.42 (118 %) | <1.01 | <1.08 | <1.08 | <1.00 | <1.08 | <1.08 | <1.00 | <1.08 | <1.08 | <1.00 | <1.08 | <1.08 |
| PFHxS | 32% | 1 | 15.4 | 17.66 (115 %) | 18.4 (120 %) | <1.01 | <1.09 | <1.09 | 3.36 | 3.88 (116 %) | 3.76 (112 %) | 2.99 | 3.9 (130 %) | 4.08 (137 %) | <1.00 | <1.09 | <1.09 |
| PFHpS | 17% | 1 | <1.00 | <1.09 | <1.09 | <1.01 | <1.09 | <1.09 | <1.00 | <1.09 | <1.09 | <1.00 | <1.09 | <1.09 | <1.00 | <1.09 | <1.09 |
| PFOS | 28% | 1 | 60.3 | 49.34 (82 %) | 52.44 (87 %) | <1.01 | 0.3 | <0.21 | 8.74 | 5.98 (68 %) | 6.76 (77 %) | 4.2 | 3.25 (77 %) | 3.57 (85 %) | <1.00 | 0.2 | 0.2 |
| PFNS | 38% | 1 | <1.00 | <1.08 | <1.08 | <1.01 | <1.08 | <1.08 | <1.00 | <1.08 | <1.08 | <1.00 | <1.08 | <1.08 | <1.00 | <1.08 | <1.08 |
| PFDS | 28% | 1 | <1.00 | <1.08 | <1.08 | <1.01 | <1.08 | <1.08 | <1.00 | <1.08 | <1.08 | <1.00 | <1.08 | <1.08 | <1.00 | <1.08 | <1.08 |
| PFUnDS | 20% | 1 | <1.00 | <1.02 | <1.02 | <1.01 | <1.02 | <1.02 | <1.00 | <1.02 | <1.02 | <1.00 | <1.02 | <1.02 | <1.00 | <1.02 | <1.02 |
| PFDoDS | 32% | 1 | <1.00 | <0.2 | <0.2 | 1.12 | <0.2 | <0.2 | <1.00 | <0.2 | <0.2 | <1.00 | <0.2 | <0.2 | <1.00 | <0.2 | <0.2 |
| PFTrDS | 33% | 1 | <1.00 | <1.03 | <1.03 | <1.01 | <1.03 | <1.03 | <1.00 | <1.03 | <1.03 | <1.00 | <1.03 | <1.03 | <1.00 | <1.03 | <1.03 |
| FOSA | 40% | 1 | <1.00 | 0.7 | 0.5 | <1.01 | <0.22 | <0.22 | <1.00 | <0.22 | <0.22 | <1.00 | <0.22 | <0.22 | <1.00 | <0.22 | <0.22 |
| N-EtFOSAA | 45% | 1 | <1.00 | <1.88 | <1.88 | <1.01 | <1.88 | <1.88 | <1.00 | <1.88 | <1.88 | <1.00 | <1.88 | <1.88 | <1.00 | <1.88 | <1.88 |
| N‑MeFOSAA | 45% | 1 | <1.00 | <0.22 | <0.22 | <1.01 | <0.22 | <0.22 | <1.00 | <0.22 | <0.22 | <1.00 | <0.22 | <0.22 | <1.00 | <0.22 | <0.22 |
| 8:2 diPAP | 45% | 1 | <1.00 | 0.4 | <0.22 | 1.42 | <0.22 | <0.22 | <1.00 | <0.22 | <0.22 | <1.00 | 0.3 | <0.22 | <1.00 | <0.22 | <0.22 |
| 8:2 FTUCA | 45% | 1 | <1.00 | <5.44 | <5.44 | <1.01 | <5.44 | <5.44 | <1.00 | <5.44 | <5.44 | <1.00 | <5.44 | <5.44 | <1.00 | <5.44 | <5.44 |

Table S4. Comparison of methods 1 and 2: 1) Developed method used in this study (polypropylene bottle, ammonium acetate) and analysed by our laboratory, 2) collection in polypropylene bottle from the accredited laboratory, no ammonium acetate added, analysed by our laboratory. Relative deviations were calculated as (Method1)/(Method 2)*100.

|  | M | | | Field blank of M | | | L4 | | | H3 | | | A* | | |
| --- | --- | --- | --- | --- | --- | --- | --- | --- | --- | --- | --- | --- | --- | --- | --- |
|  | Concentrations (ng.L^-1^) | | Relative deviation (%) | Concentrations (ng.L^-1^) | | Relative deviation (%) | Concentrations (ng.L^-1^) | | Relative deviation (%) | Concentrations (ng.L^-1^) | | Relative deviation (%) | Concentrations (ng.L^-1^) | | Relative deviation (%) |
|  | Method 2 | Method 1 |  | Method 2 | Method 1 |  | Method 2 | Method 1 |  | Method 2 | Method 1 |  | Method 2 | Method 1 |  |
| PFBA | 5.7 | 5.4 | 95 | <1.1 | <1.1 |  | 4.71 | 4.02 | 85 | 2.53 | 2.85 | 113 | <1.1 | <1.1 |  |
| PFPeA | 17.4 | 16.3 | 94 | <5.41 | <5.41 |  | 13.72 | 12.47 | 91 | 6.66 | 6.78 | 102 | <5.41 | <5.41 |  |
| PFHxA | 15.3 | 14.3 | 94 | <0.22 | 0.3 | Non conform | 10.19 | 8.68 | 86 | 4.66 | 4.87 | 105 | <0.22 | <0.22 |  |
| PFHpA | 4.4 | 4.7 | 107 | <1.07 | <1.07 |  | 3.54 | 3.18 | 90 | 2.44 | 2.09 | 86 | <1.07 | <1.07 |  |
| PFOA | 2.5 | 2.6 | 103 | <0.22 | <0.22 |  | 1.79 | 1.33 | 74 | 1.65 | 1.78 | 108 | <0.22 | <0.22 |  |
| PFNA | <1.08 | <1.08 |  | <1.08 | <1.08 |  | <1.08 | <1.08 |  | <1.08 | <1.08 |  | <1.08 | <1.08 |  |
| PFDA | <1.08 | <1.08 |  | <1.08 | <1.08 |  | <1.08 | <1.08 |  | <1.08 | <1.08 |  | <1.08 | <1.08 |  |
| PFUnDA | <0.22 | <0.22 |  | <0.22 | <0.22 |  | <0.22 | <0.22 |  | <0.22 | <0.22 |  | <0.22 | <0.22 |  |
| PFDoDA | <1.09 | <1.09 |  | <1.09 | <1.09 |  | <1.09 | <1.09 |  | <1.09 | <1.09 |  | <1.09 | <1.09 |  |
| PFTrDA | <1.08 | <1.08 |  | <1.08 | <1.08 |  | <1.08 | <1.08 |  | <1.08 | <1.08 |  | <1.08 | <1.08 |  |
| PFTeDA | <1.09 | <1.09 |  | <1.09 | <1.09 |  | <1.09 | <1.09 |  | <1.09 | <1.09 |  | <1.09 | <1.09 |  |
| PFHxDA | <1.08 | <1.08 |  | <1.08 | <1.08 |  | <1.08 | <1.08 |  | <1.08 | <1.08 |  | <1.08 | <1.08 |  |
| PFODA | NE | NE |  | 6.6 | 3.4 | 52 | NE | NE |  | NE | NE |  | NE | NE |  |
| HFPO-DA | <1.09 | <1.09 |  | <1.09 | <1.09 |  | <1.09 | <1.09 |  | <1.09 | <1.09 |  | <1.09 | <1.09 |  |
| DONA | <0.22 | <0.22 |  | <0.22 | <0.22 |  | <0.22 | <0.22 |  | <0.22 | <0.22 |  | <0.22 | <0.22 |  |
| 6:2 FTS | NE | NE |  | 100.7 | 102.7 | 102 | NE | NE |  | NE | NE |  | NE | NE |  |
| 8:2 FTS | NE | NE |  | 0.6 | 0.57 | 93 | NE | NE |  | NE | NE |  | NE | NE |  |
| 6:2 Cl-PFESA | <0.21 | <0.21 |  | <0.21 | <0.21 |  | <0.21 | <0.21 |  | <0.21 | <0.21 |  | <0.21 | <0.21 |  |
| PFBS | 2.1 | 2.1 | 101 | <1.1 | <1.1 |  | 1.4 | 1.4 | 99 | 1.57 | 1.56 | 99 | <1.1 | <1.1 |  |
| PFPeS | 2.4 | 2.4 | 103 | <1.08 | <1.08 |  | <1.08 | <1.08 |  | <1.08 | <1.08 |  | <1.08 | <1.08 |  |
| PFHxS | 17.7 | 18.4 | 104 | <1.09 | <1.09 |  | 3.88 | 3.76 | 97 | 3.9 | 4.08 | 105 | <1.09 | <1.09 |  |
| PFHpS | <1.09 | <1.09 |  | <1.09 | <1.09 |  | <1.09 | <1.09 |  | <1.09 | <1.09 |  | <1.09 | <1.09 |  |
| PFOS | 49.3 | 52.4 | 106 | 0.3 | <0.21 | Non conform | 5.98 | 6.76 | 113 | 3.25 (77.4%) | 3.57 (85%) | 110 | 0.2 | 0.2 | 110 |
| PFNS | <1.08 | <1.08 |  | <1.08 | <1.08 |  | <1.08 | <1.08 |  | <1.08 | <1.08 |  | <1.08 | <1.08 |  |
| PFDS | <1.08 | <1.08 |  | <1.08 | <1.08 |  | <1.08 | <1.08 |  | <1.08 | <1.08 |  | <1.08 | <1.08 |  |
| PFUnDS | <1.02 | <1.02 |  | <1.02 | <1.02 |  | <1.02 | <1.02 |  | <1.02 | <1.02 |  | <1.02 | <1.02 |  |
| PFDoDS | <0.2 | <0.2 |  | <0.2 | <0.2 |  | <0.2 | <0.2 |  | <0.2 | <0.2 |  | <0.2 | <0.2 |  |
| PFTrDS | <1.03 | <1.03 |  | <1.03 | <1.03 |  | <1.03 | <1.03 |  | <1.03 | <1.03 |  | <1.03 | <1.03 |  |
| FOSA | 0.7 | 0.5 | 78 | <0.22 | <0.22 |  | <0.22 | <0.22 |  | <0.22 | <0.22 |  | <0.22 | <0.22 |  |
| N-EtFOSA | <1.1 | <LQ |  | <1,1 | <1,1 |  | <1,1 | <1,1 |  | <1,1 | <1,1 |  | <1,1 | <1,1 |  |
| N-MeFOSA | <1.08 | <LQ |  | <1,08 | <1,08 |  | <1,08 | <1,08 |  | <1,08 | <1,08 |  | <1,08 | <1,08 |  |
| N-EtFOSAA | <1.88 | <1.88 |  | <1.88 | <1.88 |  | <1.88 | <1.88 |  | <1.88 | <1.88 |  | <1.88 | <1.88 |  |
| N‑MeFOSAA | <0.22 | <0.22 |  | <0.22 | <0.22 |  | <0.22 | <0.22 |  | <0.22 | <0.22 |  | <0.22 | <0.22 |  |
| 8:2 diPAP | 0.4 | <0.22 | Non conform | <0.22 | <0.22 |  | <0.22 | <0.22 |  | 0.3 | <0.22 | Non conform | <0.22 | <0.22 |  |
| 8:2 FTUCA | <5.44 | <5.44 |  | <5.44 | <5.44 |  | <5.44 | <5.44 |  | <5.44 | <5.44 |  | <5.44 | <5.44 |  |

Table S5. Individual PFAS concentration (ng.L^-1^) for the 30 samples, DET = detected (when blank percentage between 25% and 50% of the signal), < LQ = below the limit of quantification, ND = non detected, NE = non exploitable (when blank percentage > 50% of the signal).

| Compound | **A*** | **B** | **C** | **D*** | **E1** | **E2** | **E3** | **F** | **G1** | **G2** | **H1** | **H2** | **H3** | **I-TW*** | **I-GW*** | **J*** | **K1** | **K2** | **K3** | **K4** | **K5** | **L1** | **L2** | **L3** | **L4** | **M** | **N1** | **N2** | **O-TW*** | **O-GW*** |
| --- | --- | --- | --- | --- | --- | --- | --- | --- | --- | --- | --- | --- | --- | --- | --- | --- | --- | --- | --- | --- | --- | --- | --- | --- | --- | --- | --- | --- | --- | --- |
| PFBA | ND | ND | <LQ | ND | NE | <LQ | <LQ | <LQ | DET | 2.12 | DET | NE | 2.85 | NE | NE | NE | 2.61 | 2.85 | 3.91 | 2.17 | 2.47 | 5.79 | DET | DET | 4.02 | 5.41 | DET | 6.74 | DET | NE |
| PFPeA | ND | ND | ND | ND | ND | ND | ND | ND | <LQ | 5.45 | 8.11 | 6.94 | 6.78 | ND | ND | ND | <LQ | <LQ | 6.41 | <LQ | <LQ | 17.7 | 14.6 | 12.1 | 12.5 | 16.3 | 15.2 | 16 | ND | ND |
| PFHxA | <LQ | <LQ | DET | <LQ | DET | 0.715 | 0.637 | 0.759 | 3.57 | 3.59 | 6.5 | 4.82 | 4.87 | NE | NE | NE | 3.72 | 3.21 | 3.98 | 2.87 | 3.01 | 15.4 | 11.8 | 9.08 | 8.68 | 14.3 | 11.1 | 11.2 | 1.48 | 1.6 |
| PFHpA | ND | ND | ND | ND | ND | ND | ND | ND | 1.43 | 1.38 | 2.16 | 1.76 | 2.09 | ND | ND | ND | 1.39 | <LQ | ND | 1.43 | ND | 4.33 | 3.36 | 2.8 | 3.18 | 4.71 | 4.25 | 4.23 | ND | ND |
| PFOA | <LQ | <LQ | 0.576 | <LQ | DET | 0.268 | 0.295 | 0.537 | DET | 1.04 | 2.23 | 1.75 | 1.78 | NE | NE | 0.724 | 0.966 | 0.752 | 0.634 | 0.669 | 0.759 | 2.07 | 2.32 | DET | 1.33 | 2.6 | DET | 1.82 | NE | NE |
| PFNA | ND | ND | ND | ND | ND | ND | ND | ND | ND | ND | ND | ND | ND | ND | ND | ND | ND | ND | ND | ND | ND | ND | ND | ND | ND | ND | ND | NE | ND | ND |
| PFDA | <LQ | <LQ | ND | ND | ND | ND | ND | ND | ND | ND | <LQ | ND | ND | ND | ND | <LQ | <LQ | ND | ND | ND | ND | <LQ | <LQ | <LQ | ND | ND | ND | NE | ND | ND |
| PFUnDA | <LQ | <LQ | <LQ | <LQ | <LQ | <LQ | <LQ | ND | ND | ND | <LQ | <LQ | <LQ | ND | <LQ | ND | <LQ | ND | ND | ND | ND | ND | <LQ | ND | <LQ | <LQ | <LQ | NE | <LQ | <LQ |
| PFDoDA | <LQ | <LQ | ND | ND | <LQ | ND | ND | ND | <LQ | <LQ | <LQ | <LQ | ND | <LQ | <LQ | <LQ | <LQ | ND | ND | ND | ND | <LQ | <LQ | <LQ | ND | ND | <LQ | <LQ | <LQ | <LQ |
| PFTrDA | <LQ | <LQ | <LQ | <LQ | <LQ | <LQ | <LQ | <LQ | <LQ | <LQ | <LQ | <LQ | <LQ | 1.08 | <LQ | <LQ | <LQ | <LQ | <LQ | <LQ | <LQ | <LQ | <LQ | <LQ | <LQ | <LQ | <LQ | NE | <LQ | <LQ |
| PFTeDA | <LQ | <LQ | ND | <LQ | <LQ | ND | ND | ND | <LQ | ND | <LQ | <LQ | ND | <LQ | <LQ | <LQ | <LQ | <LQ | <LQ | <LQ | <LQ | <LQ | <LQ | <LQ | ND | ND | <LQ | <LQ | <LQ | <LQ |
| PFHxDA | ND | ND | ND | ND | ND | ND | ND | ND | <LQ | ND | 1.17 | <LQ | ND | <LQ | <LQ | <LQ | <LQ | ND | ND | <LQ | <LQ | <LQ | <LQ | <LQ | ND | ND | <LQ | 10.2 | <LQ | 0 |
| PFODA | NE | NE | NE | NE | 2.3 | NE | NE | NE | 2.34 | 2.56 | 2.38 | <LQ | NE | 2.33 | 2.25 | 2.48 | 2.54 | <LQ | <LQ | <LQ | <LQ | 2.73 | 2.36 | 2.59 | NE | NE | 2.22 | NE | NE | NE |
| PFBS | <LQ | <LQ | 1.23 | <LQ | ND | <LQ | <LQ | <LQ | <LQ | 1.24 | <LQ | <LQ | 1.56 | ND | ND | ND | <LQ | <LQ | <LQ | <LQ | <LQ | <LQ | <LQ | <LQ | 1.41 | 2.14 | 1.35 | NE | 1.54 | 1.49 |
| PFPeS | ND | <LQ | <LQ | ND | NE | ND | ND | <LQ | NE | <LQ | NE | NE | <LQ | NE | NE | NE | <LQ | <LQ | <LQ | <LQ | <LQ | NE | NE | NE | <LQ | 2.42 | NE | NE | NE | NE |
| L-PFHxS | ND | <LQ | <LQ | <LQ | <LQ | <LQ | <LQ | 1.19 | 1.29 | 1.82 | 2.65 | 2.46 | 3.1 | ND | ND | ND | 0.964 | <LQ | <LQ | <LQ | <LQ | 3.55 | 3.11 | 2.67 | 2.95 | 14.9 | 2.92 | NE | 1.02 | 1.08 |
| Br-PFHxS | ND | <LQ | 0.317 | 0.276 | ND | ND | ND | 0.41 | 0.365 | 0.512 | 0.737 | 0.629 | 0.978 | ND | ND | ND | 0.271 | 0.256 | <LQ | <LQ | <LQ | 0.843 | 0.812 | 0.689 | 0.803 | 3.46 | 0.803 | NE | 0.321 | 0.361 |
| PFHpS | <LQ | <LQ | <LQ | <LQ | ND | <LQ | <LQ | <LQ | <LQ | <LQ | <LQ | <LQ | <LQ | ND | ND | ND | ND | ND | ND | ND | ND | <LQ | <LQ | <LQ | <LQ | <LQ | <LQ | NE | ND | ND |
| L-PFOS | 0.181 | ND | NE | <LQ | 0.145 | DET | NE | DET | 2.15 | 2.78 | 1.5 | 1.43 | 1.87 | <LQ | <LQ | <LQ | 0.356 | 0.361 | 0.346 | 0.305 | 0.344 | 7.88 | 6.53 | 4.13 | 4.43 | 36.9 | 2.33 | NE | <LQ | <LQ |
| Br-PFOS | <LQ | <LQ | 0.703 | <LQ | 0.205 | <LQ | <LQ | 1.02 | 0.869 | 1.26 | 1.28 | 1.24 | 1.71 | ND | ND | ND | 0.52 | 0.473 | 0.505 | 0.503 | 0.49 | 3.24 | 2.72 | 1.97 | 2.33 | 15.5 | 1.27 | NE | ND | ND |
| PFNS | ND | ND | ND | ND | ND | ND | ND | ND | ND | ND | ND | ND | ND | <LQ | ND | ND | ND | ND | ND | ND | ND | ND | ND | ND | ND | <LQ | ND | NE | ND | ND |
| PFDS | ND | ND | ND | ND | <LQ | ND | <LQ | ND | <LQ | ND | <LQ | <LQ | ND | <LQ | <LQ | <LQ | ND | ND | ND | ND | ND | <LQ | <LQ | <LQ | ND | ND | <LQ | 1.28 | <LQ | <LQ |
| PFUnDS | ND | ND | ND | ND | ND | ND | ND | ND | ND | ND | ND | ND | ND | ND | ND | ND | ND | ND | ND | ND | ND | ND | ND | ND | ND | ND | ND | <LQ | ND | ND |
| PFDoDS | ND | ND | ND | ND | ND | ND | ND | ND | ND | ND | ND | ND | ND | ND | ND | ND | ND | ND | ND | ND | ND | ND | ND | ND | ND | ND | ND | ND | ND | ND |
| PFTrDS | ND | ND | ND | ND | ND | ND | ND | ND | ND | ND | ND | ND | ND | <LQ | <LQ | ND | ND | ND | ND | ND | ND | ND | ND | ND | ND | ND | ND | <LQ | ND | ND |
| HFPO-DA | ND | ND | ND | ND | ND | ND | ND | ND | ND | ND | ND | ND | ND | ND | ND | ND | ND | ND | ND | ND | ND | ND | ND | ND | ND | ND | ND | <LQ | ND | ND |
| DONA | <LQ | <LQ | <LQ | <LQ | <LQ | <LQ | <LQ | <LQ | <LQ | <LQ | <LQ | <LQ | <LQ | <LQ | <LQ | <LQ | <LQ | <LQ | <LQ | <LQ | <LQ | <LQ | <LQ | <LQ | <LQ | <LQ | <LQ | <LQ | <LQ | <LQ |
| 6:2 Cl-PFESA | <LQ | <LQ | <LQ | <LQ | ND | <LQ | <LQ | <LQ | ND | <LQ | ND | ND | <LQ | <LQ | <LQ | ND | ND | ND | ND | ND | ND | ND | ND | ND | <LQ | <LQ | ND | NE | ND | ND |
| 6:2 FTS | NE | NE | NE | NE | NE | NE | NE | NE | NE | NE | NE | NE | NE | NE | NE | NE | NE | NE | NE | NE | NE | 62.8 | 49.5 | DET | NE | NE | NE | NE | NE | NE |
| 8:2 FTS | NE | NE | NE | NE | ND | NE | NE | NE | ND | NE | ND | ND | NE | ND | ND | ND | ND | ND | ND | ND | ND | ND | ND | ND | NE | NE | ND | NE | ND | ND |
| FOSA | ND | ND | ND | ND | ND | ND | ND | ND | ND | ND | ND | ND | ND | ND | ND | ND | ND | ND | ND | ND | ND | <LQ | <LQ | ND | ND | 0.512 | ND | ND | ND | ND |
| N-EtFOSA | ND | ND | ND | ND | ND | ND | ND | ND | ND | ND | ND | ND | ND | ND | ND | ND | ND | ND | ND | ND | ND | ND | ND | ND | ND | ND | ND | ND | ND | ND |
| N-MeFOSA | ND | ND | ND | ND | ND | ND | ND | ND | ND | ND | ND | ND | ND | ND | ND | ND | ND | ND | ND | ND | ND | ND | ND | ND | ND | ND | ND | ND | ND | ND |
| L-N‑EtFOSAA | ND | ND | ND | ND | ND | ND | ND | ND | ND | ND | ND | ND | ND | ND | ND | ND | ND | ND | ND | ND | ND | ND | ND | ND | ND | ND | ND | ND | ND | ND |
| Br-N‑EtFOSAA | ND | ND | ND | ND | ND | ND | ND | ND | ND | ND | ND | ND | ND | ND | ND | ND | ND | ND | ND | ND | ND | ND | ND | ND | ND | ND | ND | ND | ND | ND |
| N‑MeFOSAA | <LQ | <LQ | <LQ | <LQ | <LQ | <LQ | <LQ | <LQ | <LQ | <LQ | <LQ | <LQ | <LQ | <LQ | <LQ | <LQ | <LQ | <LQ | <LQ | <LQ | <LQ | <LQ | <LQ | <LQ | <LQ | <LQ | <LQ | NE | <LQ | <LQ |
| 8:2 diPAP | <LQ | <LQ | <LQ | <LQ | NE | <LQ | <LQ | <LQ | NE | <LQ | NE | <LQ | <LQ | NE | NE | 0.84 | ND | ND | ND | ND | ND | ND | ND | NE | <LQ | <LQ | ND | DET | 0.222 | <LQ |
| 8:2 FTUCA | ND | ND | ND | ND | ND | ND | ND | ND | ND | ND | ND | ND | ND | ND | ND | ND | ND | ND | ND | ND | ND | ND | ND | ND | ND | ND | ND | ND | ND | ND |
| Sum of total PFAS | 0.181 | <LQ | 2.83 | 0.276 | 2.65 | 0.983 | 0.932 | 3.92 | 12 | 23.8 | 28.7 | 21 | 27.6 | 3.42 | 2.25 | 4.05 | 13.3 | 7.9 | 15.8 | 7.95 | 7.07 | 126 | 97.2 | 36 | 41.6 | 119 | 41.4 | 51.4 | 4.58 | 4.53 |
| **Sum of 20 PFAS** | 0.181 | <LQ | 2.83 | 0.276 | 0.35 | 0.983 | 0.932 | 3.92 | 9.67 | 21.2 | 25.2 | 21 | 27.6 | 1.08 | <LQ | 0.724 | 10.8 | 7.9 | 15.8 | 7.95 | 7.07 | 60.8 | 45.3 | 33.4 | 41.6 | 119 | 39.2 | 41.3 | 4.36 | 4.53 |

Figure S1. Comparison of average total PFAS concentrations (ng.L^-1^) between water catchments. A to O: Catchment identity. * Indicate drinking water supplied by the private catchments. For the location of sampling sites, see legend of Figure 1.

Table S6. Estimated Weekly Intake (EWI) values (ng.kg_bw_ ^-1^) for adults and children using an average adult body weight of 60 kg (25 kg for children), a daily drinking water intake of 2 L and an assumed 20% contribution of water to total PFAS exposure. Samples exceeding the TWI of 4,4 ng.kg_bw_ ^-1^ are highlighted in orange.

| Samples | EWI (adults) | EWI (children) |
| --- | --- | --- |
| A* | 0.211 | 0.506 |
| B | 0 | 0 |
| C | 1.86 | 4.47 |
| D* | 0.322 | 0.772 |
| E1 | 0.408 | 0.979 |
| E2 | 0.313 | 0.752 |
| E3 | 0.344 | 0.827 |
| F | 3.68 | 8.84 |
| G1 | 5.45 | 13.1 |
| G2 | 8.65 | 20.8 |
| H1 | 9.80 | 23.5 |
| H2 | 8.76 | 21.0 |
| H3 | 11.0 | 26.4 |
| I-GW* | 0 | 0 |
| I-TW* | 0 | 0 |
| J* | 0.844 | 2.03 |
| K1 | 3.59 | 8.62 |
| K2 | 2.15 | 5.16 |
| K3 | 1.73 | 4.16 |
| K4 | 1.72 | 4.13 |
| K5 | 1.86 | 4.46 |
| L1 | 20.5 | 49.2 |
| L2 | 18.1 | 43.4 |
| L3 | 11.0 | 26.5 |
| L4 | 13.8 | 33.2 |
| M | 85.7 | 206 |
| N1 | 8.54 | 20.5 |
| N2 | 2.12 | 5.09 |
| O-GW* | 1.68 | 4.03 |
| O-TW* | 1.56 | 3.76 |
